# Review of the genus *Tylopus* Jeekel, 1968, with descriptions of five new species from Thailand (Diplopoda, Polydesmida, Paradoxosomatidae)

**DOI:** 10.3897/zookeys.72.744

**Published:** 2010-12-17

**Authors:** Natdanai Likhitrakarn, Sergei I. Golovatch, Rujiporn Prateepasen, Somsak Panha

**Affiliations:** 1 Animal Systematics Research Unit, Department of Biology, Faculty of Science, Chulalongkorn University, Bangkok, 10330, Thailand; 2 Institute for Problems of Ecology and Evolution, Russian Academy of Sciences, Leninsky pr. 33, Moscow119071, Russia; 3 The Scientific and Technological Research Equipment Center, Chulalongkorn University,Bangkok, 10330, Thailand.

**Keywords:** millipede, *Tylopus*, taxonomy, new species, key, Thailand

## Abstract

The genus Tylopus currently contains 41 species, all keyed and mapped, including five new from northern Thailand: Tylopus bispinosus **sp. n.**, Tylopus grandis **sp. n.**, Tylopus extremus **sp. n.**, Tylopus veliger **sp. n.** and Tylopus parajeekeli **sp. n.** Species of Tylopus are predominantly forest-dwellers, especially in montane habitats where up to 9–10 species can coexist per faunule. We expect many more congeners to be discovered in future, in particular from poorly or relatively poorly prospected regions such as Laos (only two species recorded), Cambodia (no species yet), Vietnam (a few species), Myanmar (a few species) and southern China (one species only). Because the genus is so species-rich and as yet so poorly sampled, a phylogenetic analysis of Tylopus would be premature.

## Introduction

Tylopus Jeekel, 1968, is one of the largest and most common genera in the millipede family Paradoxosomatidae in Southeast Asia and adjacent parts of southern China. When last reviewed (Golovatch and Enghoff 1993), it comprised 35 species. Only one more has since been added (Golovatch 1995), thus bringing the number of known species to 36.

The present paper provides a new review of Tylopus, based on numerous recently collected samples which also include five new species from northern Thailand. These new species are described herein, another seven are redescribed based on additional samples, and a new key is provided to incorporate all 41 species currently known to comprise Tylopus. We are confident that many more species in this genus will be found in future, given that several large areas in Laos, Myanmar, Cambodia and Vietnam, as well as in southern China, are as yet poorly sampled for millipedes. At present, perhaps only Thailand can be regarded as relatively well prospected, and has already yielded 26 Tylopus species.

## Material and methods

New material derives from several provinces of northern Thailand taken between 2006 and 2010. All holotypes, as well as most of the paratypes and non-types, are in the collection of the Museum of Zoology, Chulalongkorn University, Bangkok, Thailand (CUMZ), some duplicates also being donated to the collections of the Natural History Museum of Denmark, University of Copenhagen, Denmark (ZMUC), and of the Zoological Museum, State University of Moscow, Russia (ZMUM), as indicated in the text.

Coloration was photographed in the laboratory (both live and alcohol material) for all of the encountered species. Material was then fixed, preserved in 75% ethanol and studied in the lab using a standard Olympus stereomicroscope. Scanning electron micrographs (SEM) were taken using a JEOL, JSM-5410 LV microscope. After SEM examination of the gonopods, they were returned to alcohol.

## Taxonomic part

### Checklist

The following species of Tylopus have heretofore been described, all arranged in alphabetic order and supplied with geographic details:

Tylopus affinis Golovatch and Enghoff 1993 – Thailand, Chiang Mai Province, Doi Suthep National Park, Doi Pui summit, 1650 m; Doi Inthanon National Park, Mae Chaem road, 1700 m; same locality, main road, 1900 m.

Tylopus allorugosus Golovatch and Enghoff 1993 – Thailand, Chiang Mai Province, Doi Inthanon National Park, SiriphumWaterfall, 1300–1400 m; same locality, ca 1600 m; same locality, Mae Chaem road, 1700 m; same locality, main road, 1900 m; same locality, main road, 2200 m; same locality, 2200–2500 m; Doi Suthep National Park, Doi Pui summit, 1650 m.

Tylopus amicus Golovatch and Enghoff 1993 – Thailand, Chiang Mai Province, Doi Pha Hom Pok National Park, northwest of Fang, 1550–1750 m.

Tylopus asper Golovatch and Enghoff 1993 – Thailand, Chiang Mai Province, Doi Inthanon National Park, 1500 m.

Tylopus baenzigeri Golovatch and Enghoff 1993 – Thailand, Chiang Mai Province, Doi Suthep National Park, Doi Pui-Chang Khian, 1400 m; same locality, 1400–1500 m; Doi Suthep National Park, near stream, 1100 m.

Tylopus coriaceus Golovatch and Enghoff 1993 – Thailand, Chaiyaphum Province, Khon San District, Phu Kheio, 16°22', 101°34', 1000 m.

Tylopus crassipes Golovatch 1984 – Vietnam, Lao cai Province, O quy ho, near Sa pa, 1900 m; same locality, near stream, 1950 m.

Tylopus degerboelae Golovatch and Enghoff 1993 – Thailand, Chiang Mai Province, Doi Suthep National Park, forest near stream, 1000 m; same locality, Doi Pui road, 1000 m; same locality, 1100 m; same locality, evergreen forest, 1300 m; same locality, evergreen forest, 1400 m; same locality, 1450 m; same locality, 1500 m; Doi Inthanon National Park, 1500 m; same locality, main road, 1600 m; Doi Chiang Dao, limestone area.

Tylopus doriae (Pocock 1895) – east-central Myanmar, Yado, 1000–1400 m, Bia-po, 1000–1200 m, Meteleo, 900–1200 m; Puepoli, 900–1200 m; Thailand, Chiang Mai Province, Doi Suthep National Park, 1400–1500 m.

Tylopus granulatus Golovatch 1984 – Vietnam, Ninh binh Province, Cuc Phuong Nature Reserve, forest.

Tylopus haplorugosus Golovatch and Enghoff 1993 – Thailand, Chiang Mai Province, Doi Inthanon National Park, main road, 1900 m.

Tylopus hilaris (Attems 1937) – Vietnam, Bana, 1500 m.

Tylopus hilaroides Golovatch 1984 – Vietnam, Ninh binh Province, Cuc Phuong Nature Reserve, forest.

Tylopus hoffmani Golovatch and Enghoff 1993 – Thailand, Chiang Mai Province, Doi Suthep, summit, 1600 m.

Tylopus jeekeli Golovatch and Enghoff 1993 – Thailand, Chiang Mai Province, Doi Inthanon National Park, Siriphum Waterfall, 1200–1300 m.

Tylopus maculatus Golovatch 1984 – Vietnam, Lao cai Province, O quy ho, near Sa pa, 1950 m.

Tylopus magicus Golovatch 1984 – Vietnam, Lao cai Province, O quy ho, near Sa pa, 1950 m.

Tylopus mutilatus (Attems 1953) – Laos, Luang Prabang; Xieng Kuang; Vietnam, Lam Dong Province, Peak Langbiang.

Tylopus nodulipes (Attems 1953) – Laos, Luang Prabang; Vietnam, Lao cai Province, Mt Fan-Si-Pan.

Tylopus pallidus Golovatch and Enghoff 1993 – Thailand, Chiang Mai Province, Doi Pha Hom Pok, northwest of Fang, 1550–1750 m.

Tylopus perarmatus Hoffman 1973 – Thailand, Chiang Mai Province, Doi Suthep National Park, east slope, 1100–1275 m; same locality, 1000 m, same locality, 1100 m; same locality, Mahidol Waterfall, 1250 m; same locality, 1400–1500 m; ca 10 miles west of Chiang Mai; Doi Inthanon National Park, Siriphum Waterfall, 1300–1400 m; same locality, Vajirathan Waterfall, 750 m; Doi Chiang Dao, ca 500 m; same locality, limestone cave; Lampang Province, Thoen District, ca 8 km east of Ban Huai Kaeo, sandy bank of stream, 900 m.

Tylopus perplexus Golovatch and Enghoff 1993 – Thailand, Chiang Mai Province, Doi Pha Hom Pok, northwest of Fang, 1550–1750 m.

Tylopus poolpermorum Golovatch and Enghoff 1993 – Thailand, Chiang Mai Province, Doi Pha Hom Pok, northwest of Fang, 1550–1750 m.

Tylopus procurvus Golovatch 1984 – Vietnam, Lao cai Province, O quy ho, pass between Lao cai and Lai chau provinces, 2160 m; same locality, O quy ho, near Sa pa, near stream, 1950 m.

Tylopus prosperus Golovatch and Enghoff 1993 – Thailand, Chiang Mai Province, Doi Inthanon National Park, main road, 2200 m; same locality, summit, 2500 m.

Tylopus pulvinipes Golovatch and Enghoff 1993 – Thailand, Chaiyaphum Province, Phu Kheio, 16°22', 101°34', Tong Kamang Noi, forest, 1000 m.

Tylopus rugosus Golovatch and Enghoff 1993 – Thailand, Chiang Mai Province, Chiang Dao, 1800 m.

Tylopus semirugosus Golovatch and Enghoff 1993 – Thailand, Tak Province, Mae Sot District, Ban Mussoe.

Tylopus sigma (Attems 1953) – Vietnam, Lao cai Province, Sa pa.

Tylopus silvestris (Pocock 1895) – Myanmar, village of Thao (Carin Ghecu), 1200–1400 m.

Tylopus similirugosus Golovatch and Enghoff 1993 – Thailand, Chiang Mai Province, Doi Suthep National Park, 1000 m; same locality, 1400–1500 m.

Tylopus sinensis Golovatch 1995 – China, Yunnan Province, Mengzi County, Pot Hole No. 2 (Ha Fa Tiao Dong).

Tylopus strongylosomoides (Korsós and Golovatch 1989) – Vietnam, Vinh phu Province, Tam Dao, north of the village.

Tylopus subcoriaceus Golovatch and Enghoff 1993 – Thailand, Chiang Mai Province, Doi Suthep National Park, near stream, 1000 m; same locality, evergreen forest, 1100 m.

Tylopus tamdaoensis Korsós and Golovatch 1989 – Vietnam, Vinh phu Province, Tam Dao, north of the village; same locality, subtropical rain forest, ca 800–1200 m.

Tylopus topali Golovatch 1984 – Vietnam, Ninh binh Province, Cuc Phuong Nature Reserve.

### Gonopod structure

Tylopus is known to be defined, among other characters, by its relatively elaborate gonopod conformation, sometimes perhaps amongst the most complex not only in the tribe Sulciferini it belongs to, but also in the Paradoxosomatidae as a whole. Even though a thorough, still fully valid review of gonopod structure is available (Golovatch and Enghoff 1993), we feel tempted to reiterate here the main morphological terms before describing new species and providing some descriptive notes concerning already known congeners.

The gonopod telopodite in Tylopus usually shows a distinct transverse ring, or cingulum, demarcating the postfemoral region which starts at the base of a free, flagelliform solenomere. The solenomere is largely sheathed by a slender and sigmoid solenophore usually bearing a number of outgrowths at its base. The cingulum is only rarely incomplete due to a somewhat reduced sulcus at the base of lobe **l**, like the one observed in Tylopus grandis sp. n. (Figs 5 and 6). Usually lobe **l** is simple, but sometimes it can be crowned with a larger (e.g. Tylopus extremus sp. n., Figs 8 and 9, or Tylopus veliger sp. n., Figs 11 and 12) or smaller outgrowth (e.g. Tylopus degerboelae, Figs 20 and 21, or Tylopus nodulipes). In addition to lobe **l**, the postfemoral region is nearly always supplied with a more or less evident process **h** lying mesally of the lobe. However, **h** is absent from Tylopus strongylosomoides. All other disto- and/or postfemoral outgrowths, based on their positions, appear to be even more optional. Thus, process **z** is mostly discernible, yet occasionally very small (e.g. Tylopus parajeekeli, Figs 14 and 15, Tylopus jeekeli, Figs 26 and 27, or Tylopus hoffmani) to fully missing (e.g. Tylopus degerboelae, Figs 20 and 21, Tylopus haplorugosus, Figs 23 and 24, or Tylopus prosperus, Figs 29 and 30). Only a few species appear to show particularly complex gonopods. Then not only does the postfemoral region bear a long, spiniform process **z**, e.g. Tylopus perarmatus (Figs 34 and 35), but also the femorite can be supplied with a small, inconspicuous, lobiform (e.g. Tylopus tamdaoensis) to very long, knife- to spine-shaped, distodorsal outgrowth **m** (Tylopus extremus sp. n., Figs 8 and 9, or Tylopus perplexus). Besides this, even a few more structures can be added to the postfemoral region, as is observed in Tylopus perplexus. It is the sizes and shapes of these various outgrowths that provide several further important species-specific characters in addition to a good number of peripheral ones (Golovatch and Enghoff 1993).

### Description of new species

#### Tylopus bispinosus sp. n.

urn:lsid:zoobank.org:act:D501889C-39AB-427B-A47D-0903412C651C

Figs 1 3 

##### Holotype

♂ (CUMZ), Thailand, Tak Province, Umphang District, near Umphang City, ca 490 m, 16°2'20N, 98°52E, 6.07.2009, leg. S. Panha, J. Sutcharit & N. Likhitrakarn.

##### Paratypes:

1 ♂, 1 ♀, 2 juv. (CUMZ), same locality, together with holotype. 6 ♂, 4 ♀ (CUMZ), 3 ♂ (ZMUC), 3 ♂ (ZMUM), Tak Province, Umphang District, Doi Hua Mod, 900 m, 16°3'14N, 98°49'16E, 5.06.2009, leg. S. Panha, J. Sutcharit & N. Likhitrakarn. 6 ♂, 1 ♀ (CUMZ), same Province, same District, Cave Ta Ko Bi, ca 530 m, 16°03'14N, 98°49'14E, 5.07.2009, leg. S. Panha, J. Sutcharit & N. Likhitrakarn.

##### Name:

To emphasize the spiniform processes **h** and **z** of the gonopod.

##### Diagnosis:

Differs from congeners in both processes **h** and **z** of the gonopod being spiniform.

##### Description:

**Figure 1.**
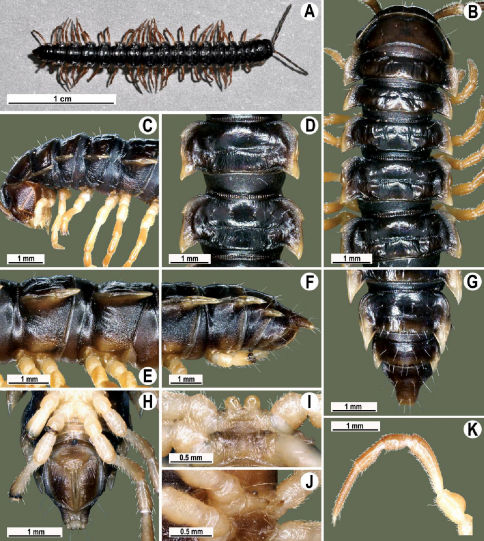
Tylopus bispinosus sp. n., ♂ paratype from near Umphang City (A) and ♂ paratype from Doi Hua Mod (B–K). **A** habitus, live coloration **B, C** anterior part of body, dorsal and lateral views, respectively. **D, E** segments 10 and 11, dorsal and lateral views, respectively **F, G, H** posterior part of body, lateral, dorsal and ventral views, respectively **I, J** sternal cones between coxae 4, subcaudal and sublateral views, respectively **K** midbody leg.

Length 26 mm (holotype), 25–29 mm (♂), 33–38 mm (♀), width of midbody pro- and metazona 2.0 and 2.9 mm (holotype), 1.8–2.4 and 3.1–3.2 mm (♂), 2.4–2.7 and 3.3–3.8 mm (♀), respectively. Coloration of live animals black-brown (Fig. 1A): calluses of paraterga, venter and legs only slightly lighter, dark brown, but turning light brown in alcohol (Fig. 1A–K).

Clypeolabral region of head very densely, vertigial region sparsely setose. Epicranial suture distinct. Antennae long and slender, reaching behind segment 4 (♂) or 3 (♀) dorsally. In width, head < collum < segments 3–4 < 2 < 5–16 (♂), or head = segment 3 < 4 < collum < segments 5–16 (♀); thereafter body gradually and gently tapering towards telson (Fig. 1B).

Tegument generally rather smooth and shining, but prozona very finely rugulose, metaterga often rugose (Fig. 1B–G); surface below paraterga finely microgranular (Fig. 1E, F). Collum with three transverse rows of setae: 5+5 in anterior, 2+2 in middle, and 4+4 in posterior row; paraterga evident, rounded, flap-shaped (Fig. 1B, C). Metaterga with two transverse rows of rather long setae: 2+2 in anterior and 2(3)+2(3) in posterior row, the latter often abraded, but then readily traceable as insertion points. Axial line at most barely visible only on metaterga. Paraterga strongly developed (Fig. 1A–G), lying high (at 1/3–1/4 midbody height), only slightly inclined laterally, pointed caudally and acutangular already from segment 2, especially strongly so on caudal segments; calluses very thin on poreless segments, slightly thicker on pore-bearing ones; anterior 1/3 of poreless calluses with two evident (anterior larger, posterior smaller), lateral, setigerous incisions, but with only a single strong one (anterior) on pore-bearing calluses (Fig. 1B–G); paraterga more strongly developed in ♂. Ozopores entirely lateral, lying in an ovoid groove about 1/3 in front of caudal corner, the latter always surpassing rear tergal contour (Fig. 1B–H). Transverse sulcus evident on metaterga 4–18, reaching base of paraterga, evident and rather deep, faintly rugulose at bottom. Stricture between pro- and metazona very clearly ribbed (Fig. 1D). Epiproct tip faintly concave to subtruncate, pre-apical papillae evident (Fig. 1G). Hypoproct roundly subtrapeziform, caudal setae strongly separated (Fig. 1H). Pleurosternal carinae well-developed on segments 2–17 (♂) or 2–7 (♀), mostly as low bulges anteriorly and a distinct spine posteriorly (Fig. 1C, E, F).

Sterna moderately setose, without modifications; a deeply notched sternal lobe between ♂coxae 4 (Fig. 1I, J). Legs long and slender (Fig. 1B, C, H), especially so in ♂ compared to ♀ (1.3–1.5 versus 0.9–1.1 times as long as midbody height); prefemora distinctly bulged laterally (Fig. 1K), acropodites with particularly dense, nearly adpressed setae, including tarsal brushes.

**Figure 2.**
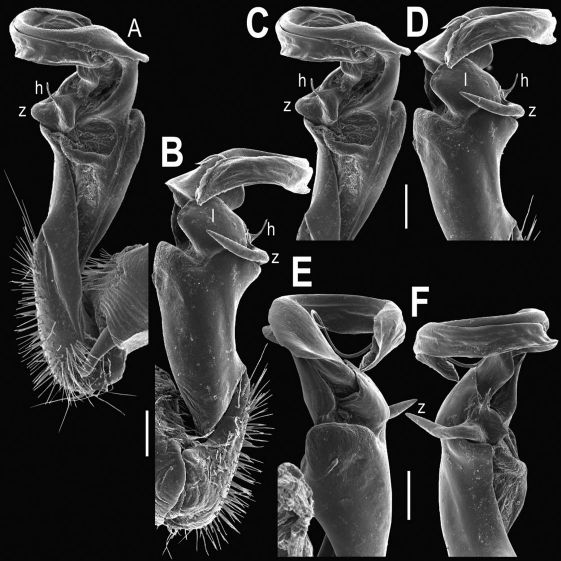
Tylopus bispinosus sp. n., ♂ paratype from Doi Hua Mod. **A, B** right gonopod, mesal and lateral views, respectively **C–F** distal part of right gonopod, mesal, lateral, subcaudal and suboral views, respectively. Scale bar: 0.2 mm.

**Figure 3.**
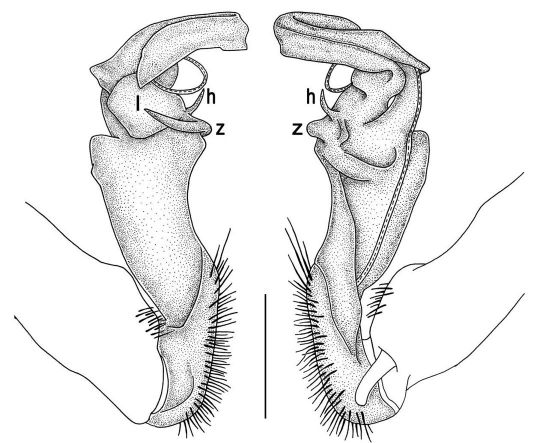
Tylopus bispinosus sp. n., ♂ paratype from Doi Hua Mod. **A, B** right gonopod, lateral and mesal views, respectively. Scale bar: 0.5 mm.

Gonopods (Figs 2, 3) with lobe **l** well-demarcated; spine **h** very small; spine **z** considerably more prominent.

#### Tylopus grandis sp. n.

urn:lsid:zoobank.org:act:148C0F96-F560-437A-92FC-6FA159B96699

Figs 4 [Fig F5] 6 

##### Holotype

♂ (CUMZ), Thailand, Mae Hong Son Province, Pangmapha District, near Cave Pha Mon, 19.07.2008, leg. S. Panha, J. Sutcharit & N. Likhitrakarn.

##### Paratypes:

1 ♂, 2 ♀ (CUMZ), same lcality, together with holotype. 1 ♂ (CUMZ), same District, Mae Lana crossroads, 19.07.2008, leg. S. Panha, J. Sutcharit & N. Likhitrakarn.

##### Name:

To emphasize the large size of this species

##### Diagnosis:

Differs from congeners in the large size, coupled with a short spiniform process **h**, a basally only poorly delimited lobe **l**, and a small lobiform process **z** of the gonopod.

##### Description:

**Figure 4.**
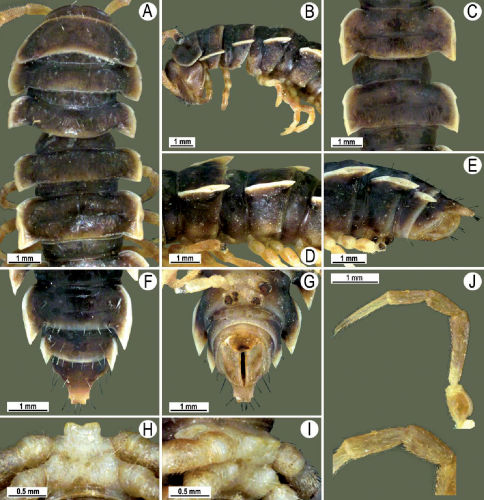
Tylopus grandis sp. n., ♂ paratype from Mae Lana (**A–J**). **A, B** anterior part of body, dorsal and lateral views, respectively **C, D** segments 10 and 11, dorsal and lateral views, respectively **E, F, G** posterior part of body, lateral, dorsal and ventral views, respectively **H, I** sternal cones between coxae 4, subcaudal and sublateral views, respectively **J** midbody leg.

Length 41 mm (holotype), 40–42 mm (♂), 38–39 mm (♀), width of midbody pro- and metazona 3.0 and 4.5 mm (holotype), 2.8–3.0 and 4.3–4.5 mm (♂), 3.6–3.8 and 4.7–5.0 mm (♀), respectively. Coloration in alcohol dark brown to black-brown (Fig. 4A–G): calluses, venter and antennomeres 1–5 slightly to considerably lighter, brown to light yellow-brown (Fig. 1A–G), antennomeres 6 and 7 dark brown.

All characters as in Tylopus bispinosus sp. n., except as follows.

Antennae short and slender (Fig. 4B), reaching behind segment 3 (♂) or 2 (♀) dorsally. In width, head <collum = segments 3–4 < 2 = 5–16 (♂), or head < segments 3–4 < collum < segment 2 < 5–16 (♀); thereafter body gradually and gently tapering towards telson (Fig. 4A–F).

Tegument generally rather smooth and either dull (only in places modestly shining) or shining (Fig. 4A–G). Paraterga strongly developed (Fig. 4A–G), lying high (at 1/4–1/5 midbody height), subhorizontal to slightly upturned laterally (Fig. 4A–F). Transverse sulcus either absent or poorly developed, then not reaching bases of paraterga 4, always evident and reaching bases of paraterga 5–18, rather faintly rugulose at bottom. Stricture between pro- and metazona rather faintly beaded to striolate (Fig. 4A–C). Epiproct tip evidently emarginate, pre-apical papillae very distinct (Fig. 4F, G). Hypoproct semi-circular, caudal setae strongly separated (Fig. 4G). Pleurosternal carinae visible on segments 2–15(16) (♂) or segments 2–6 (♀), mostly as low bulges anteriorly and a more or less distinct denticle posteriorly (Fig. 4B, D).

Sterna moderately setose, without modifications; a slightly notched sternal lobe between ♂ coxae 4 (Fig. 4H, I). Legs long and slender (Fig. 1B, C, H), especially so in ♂ compared to ♀ (1.7–1.8 versus 1.5–1.6 times as long as midbody height); ♂ prefemora distinctly bulged laterally (Fig. 4J), acropodites with particularly dense, nearly adpressed setae, but tarsal brushes missing.

**Figure 5. F5:**
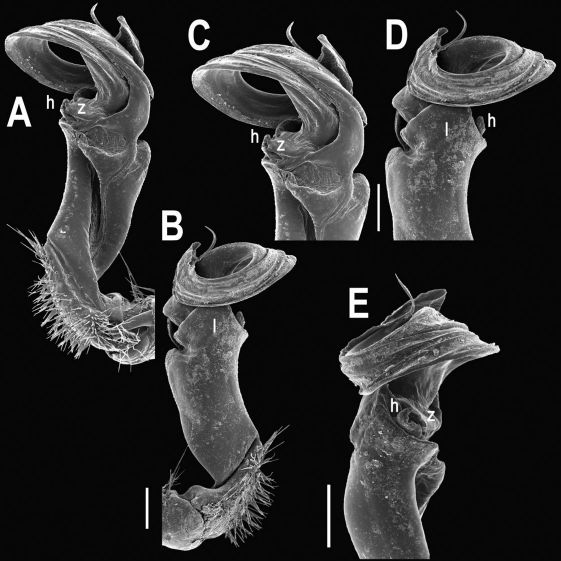
Tylopus grandis sp. n., ♂ paratype from Mae Lana. **A, B** right gonopod, mesal and lateral views, respectively **C–E** distal part of right gonopod, mesal, lateral and suboral views, respectively. Scale bar: 0.2 mm.

**Figure 6.**
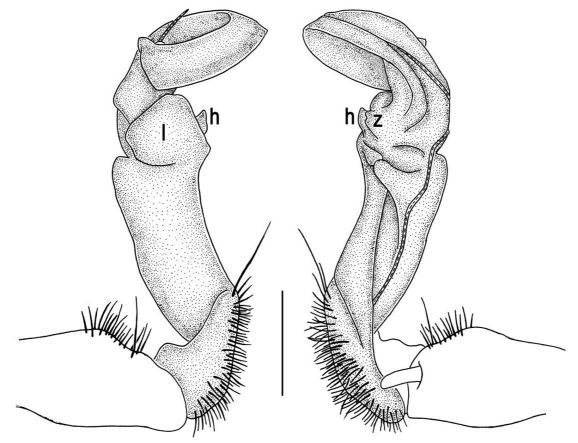
Tylopus grandis sp. n., ♂ paratype from Mae Lana. **A, B** right gonopod, lateral and mesal views, respectively. Scale bar: 0.5 mm.

Gonopods (Figs 5, 6) with lobe **l** poorly demarcated at base; spine **h** very small; process **z** not spiniform, but like a short lobe.

#### Tylopus extremus sp. n.

urn:lsid:zoobank.org:act:188F2E10-CAC7-406A-B176-6818DF526D0B

Figs 7 [Fig F8] 9 

##### Holotype

♂ (CUMZ), Thailand, Chiang Mai Province, Fang District, Doi Phahom Pok National Park, 6.07.2009, leg. A. Pansook.

##### Paratypes:

1 ♂, 1 ♀ (CUMZ), 1 ♂ (ZMUC), 1 ♂ (ZMUM), same locality, together with holotype.

##### Name:

To emphasize the extremely long spines **h** and **m** of the gonopod.

##### Diagnosis:

Differs from congeners in process **h** being subflagelliform while process **m** extremely long and prominent.

##### Description:

**Figure 7.**
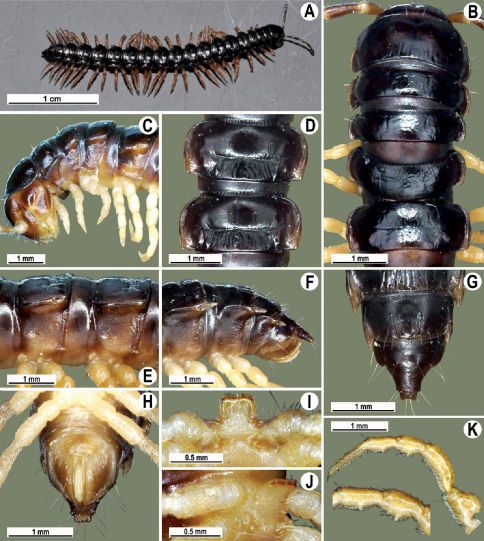
Tylopus extremus sp. n., ♂ paratype (**A–K**). **A** habitus, live coloration **B, C** anterior part of body, dorsal and lateral views, respectively **D, E** segments 10 and 11, dorsal and lateral views, respectively **F, G, H** posterior part of body, lateral, dorsal and ventral views, respectively **I, J** sternal cones between coxae 4, subcaudal and sublateral views, respectively **K** midbody leg.

Length ca 30 mm (holotype), 27–30 mm (♂), 32.5 mm (♀), width of midbody pro- and metazona 2.0 and 2.9 mm (holotype), 1.9–2.4 and 2.8–3.3 mm (♂), 2.5 and 3.0 mm (♀), respectively. Coloration of live animals, as well as of alcohol material black-brown (Fig. 7A–G): calluses of paraterga and antennae only slightly lighter, light brown to brown, venter and legs contrastingly light, yellow (Fig. 7A–G), tip of antennae pallid.

All characters as in Tylopus bispinosus sp. n., except as follows.

Antennae rather short and slender, reaching behind to end of segment 3 (♂) dorsally. Collum with paraterga like rudimentary flaps, especially poorly developed in ♀. In width, head < collum = segments 3–4 < 2 < 5–16 (♂) (Fig. 7B), or head < collum < segment 3 < 2 and 4 < 5–16(♀); thereafter body gradually and gently tapering towards telson.

Metaterga with two transverse rows of rather long setae: 2+2 in anterior and 2(3)+2(3) in posterior row, the latter often abraded, but then readily traceable as insertion points on low longitudinal ridges or tubercles (Fig. 7B–G). Axial line thin, visible on both halves of metaterga. Paraterga strongly developed (Fig. 7A–G), lying rather low (at 1/2–1/3 midbody height), slightly inclined laterally, pointed caudally and acutangular already from segment 2, especially strongly so on caudal segments, very clearly surpassing rear contour only on segments 16–19; anterior 1/3 of poreless calluses with two barely visible, lateral, setigerous incisions, but with only a single, likewise poorly developed incision anteriorly on pore-bearing calluses (Fig. 7B–F); paraterga slightly less strongly developed in ♀. Transverse sulcus evident on metaterga 5–18, reaching bases of paraterga, evident and rather deep, faintly rugulose at bottom. Stricture between pro- and metazona weakly striolate (Fig. 7B–G). Epiproct emarginate at tip, pre-apical papillae evident (Fig. 7G). Hypoproct subtrapeziform, caudal setae widely separated (Fig. 7H). Pleurosternal carinae as compete ridges with a caudal tooth on segments 2–4 (♂) or 2 and 3 (♀), like separated anterior bulges and increasingly poorly developed caudal denticles until segment 16 (Fig. 7C, E, F).

Sterna moderately setose, without modifications; an entire, linguiform, sternal lobe between ♂ coxae 4 (Fig. 7I, J). Legs long, in ♂ very distinctly incrassate, 1.7–2.0 or ca 1.3 times as long as midbody height in ♂ and ♀, respectively (Fig. 7B, C, H), ♂ prefemora very distinctly bulged laterally and clothed with dense and adpressed pilosity ventrally (Fig. 7K), acropodites also with similarly dense and ventrally adpressed pilosity, including tarsal brushes. All ♂ postfemora and tibiae except for a few posteriormost ones with a small, but evident adenostyle (= tubercle) at midway on ventral side (Fig. 7K).

**Figure 8. F8:**
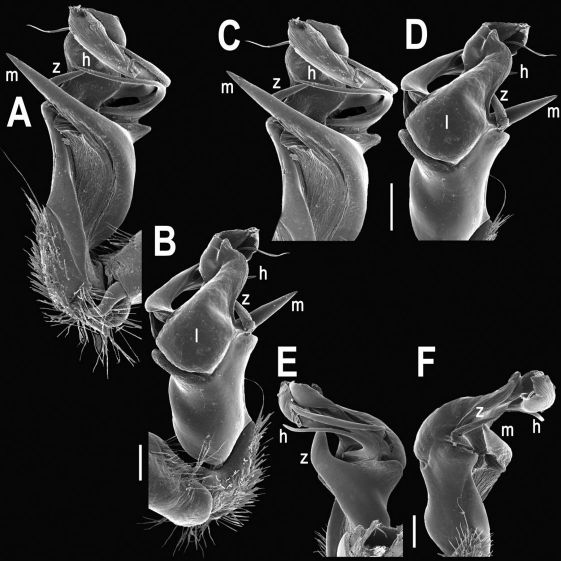
Tylopus extremus sp. n., ♂ paratype. **A, B** right gonopod, mesal and lateral views, respectively **C–F** distal part of right gonopod, mesal, lateral, subcaudal and suboral views, respectively. Scale bar: 0.2 mm.

**Figure 9.**
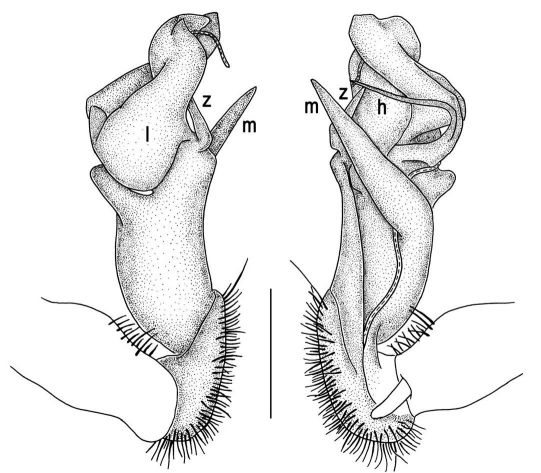
Tylopus extremus sp. n., ♂ paratype. **A, B** right gonopod, lateral and mesal views, respectively. Scale bar: 0.5 mm.

Gonopods (Figs 8, 9) with lobe **l** well-demarcated, but unusually prominent, high and elongated; spine **h** long, extremely slender and subflagelliform; spine **z** rather short and simple; spine **m** very prominent, straight and long.

#### Tylopus veliger sp. n.

urn:lsid:zoobank.org:act:54694D7D-8C76-4705-B81F-0949DFE0D787

**Figure 10.**
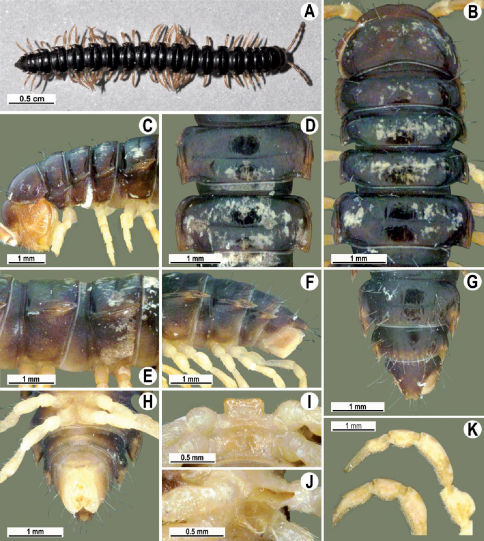
Tylopus veliger sp. n., ♂ holotype (**A–K**). **A** habitus, live coloration **B, C** anterior part of body, dorsal and lateral views, respectively **D, E** segments 10 and 11, dorsal and lateral views, respectively **F, G, H** posterior part of body, lateral, dorsal and ventral views, respectively **I, J** sternal cones between coxae 4, subcaudal and sublateral views, respectively **K** midbody leg.

Figs 10 [Fig F11] 12 

##### Holotype

♂ (CUMZ), Thailand, Nan Province, Pua District, Ton Tong Waterfall, ca 1130 m, 19°10'52N, 101°5'45E, 10.10.2009, leg. S. Panha, J. Sutcharit & N. Likhitrakarn.

##### Name:

To emphasize the velum-shaped end of gonopod lobe **l**.

##### Diagnosis:

Differs from congeners except Tylopus perplexus Golovatch and Enghoff 1993 in the distal part of gonopod lobe **l** being velum-shaped and supplied with two denticles, from Tylopus perplexus in the gonopod lacking spines **m** and **q**, as well as in a much shorter and knife-shaped spine **z**, and a rudimentary spine **h**.

##### Description:

Length ca 28 mm, width of midbody pro- and metazona 2.0 and 2.6 mm, respectively. Coloration of live animal and alcohol material rather uniformly dark brown to blackish (Fig. 1A); calluses of paraterga only slightly flavous, brown; antennomeres 1–6 and genae light brown; venter and legs contrastingly yellowish to light brown (Fig. 10A–G).

All characters as in Tylopus bispinosus sp. n., except as follows.

Clypeolabral region of head very densely setose, but vertigial region bare. Antennae short and barely reaching behind segment 2 dorsally. In width, head = segments 3 and 4 < collum < segment 2 < 5–16; thereafter body gradually and gently tapering towards telson (Fig. 10A–G). Collum with small, narrowly delimited, rounded, strip-shaped paraterga (Fig 10B, C).

Metaterga with two transverse rows of long setae: 2+2 in anterior and 2(3)+2(3) in posterior row, the latter often abraded, but then readily traceable as insertion points. Axial line thin, in places incomplete, but readily visible on both halves of metaterga. Paraterga strongly developed (Fig. 10A–G), lying relatively low (at 1/2–1/3 midbody height), evidently inclined ventrolaterally, pointed caudally and acutangular already from segment 2, especially strongly so and surpassing rear tergal contour on segments 16–19; calluses slightly thinner on poreless segments than on pore-bearing ones; poreless calluses with two lateral setigerous incisions, but with only a single, more evident one (anterior) on pore-bearing calluses (Fig. 10B–G). Transverse sulcus evident on metaterga 5–18, reaching bases of paraterga, evident and rather deep, finely, densely and clearly ribbed at bottom. Stricture between pro- and metazona very clearly ribbed (Fig. 10B–G). Epiproct tip clearly emarginate, pre-apical papillae evident (Fig. 10F–H). Hypoproct semi-circular, caudal setae strongly separated (Fig. 10H). Pleurosternal carinae as complete ridges on segments 2–4, thereafter broken into an anterior bulge and a caudal tooth, both growing increasingly reduced until segment 16 (Fig. 10C, E, F).

Sterna rather densely setose, without modifications except for a subquadrate, setose, sternal lobe between coxae 4 (Fig. 10I, J). Legs relatively short, ca 1.2–1.3 times as long as midbody height, evidently incrassate (Fig. 10C, F, K); prefemora distinctly bulged laterally and clothed with mostly adpressed setae ventrally (Fig. 10K), acropodites likewise with very dense, mostly adpressed setae ventrally; postfemora and tibiae slightly bulged ventrally; tarsal brushes missing.

**Figure 11. F11:**
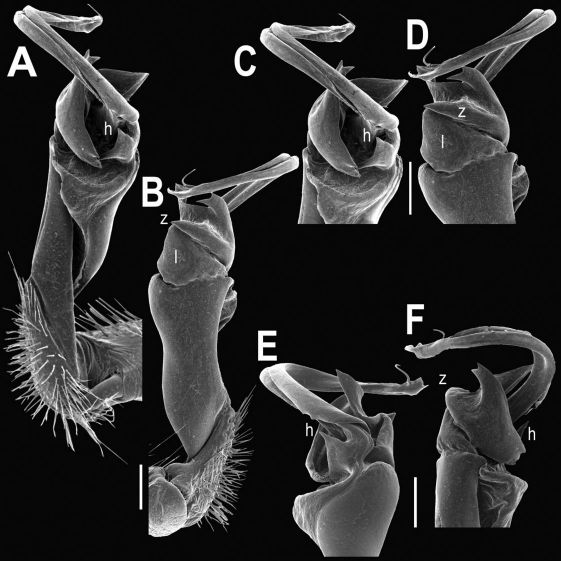
Tylopus veliger sp. n., ♂ holotype. **A, B** right gonopod, mesal and lateral views, respectively **C–F** distal part of right gonopod, mesal, lateral, subcaudal and suboral views, respectively. Scale bar: 0.2 mm.

**Figure 12.**
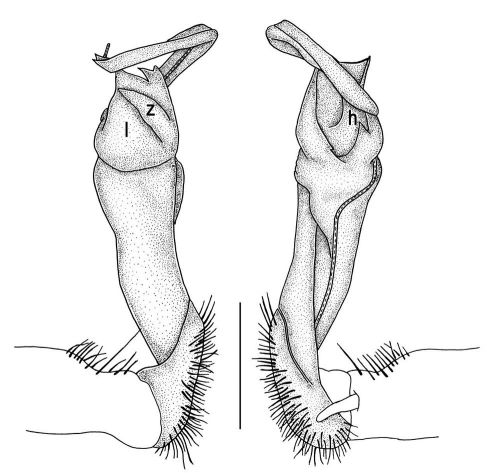
Tylopus veliger sp. n., ♂ holotype. **A, B** right gonopod, lateral and mesal views, respectively. Scale bar: 0.5 mm.

Gonopods (Figs 11, 12) with lobe **l** well-demarcated, high and prominent, apically with a pointed fan-shaped structure (= velum) and two denticles; spine **h** very small, dentiform; spine **z** prominent, knife-shaped, lying above **l** on lateral side.

#### Tylopus parajeekeli sp. n.

urn:lsid:zoobank.org:act:703DB743-0898-4B3A-8D37-A4DD31FE7CD1

**Figure 13.**
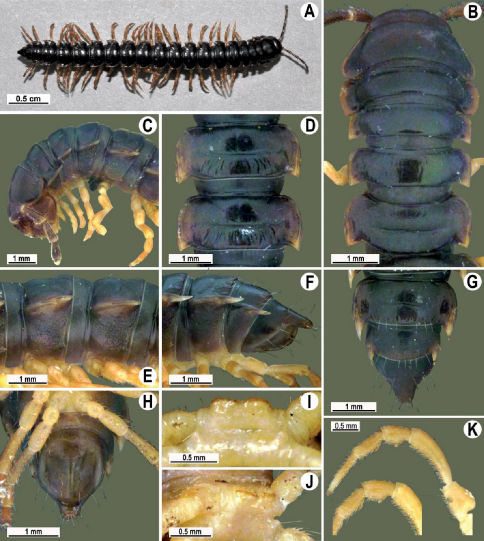
Tylopus parajeekeli sp. n., ♂ holotype (**A**) and ♂ paratype (**B–K**). **A** habitus, live coloration **B, C** anterior part of body, dorsal and lateral views, respectively **D, E** segments 10 and 11, dorsal and lateral views, respectively **F, G, H** posterior part of body, lateral, dorsal and ventral views, respectively **I, J** sternal cones between coxae 4, subcaudal and sublateral views, respectively **K** midbody leg.

Figs 13 [Fig F14] 15 

##### Holotype

♂ (CUMZ), Thailand, Chiang Mai Province, Chom Thong District, Doi Inthanon National Park, summit, 2520 m, 18°34'29N, 98°28'48E, 12.10.2009, leg. S. Panha, J. Sutcharit & N. Likhitrakarn.

##### Paratype:

1 ♂ (CUMZ), same locality, together with holotype.

##### Name:

To emphasize the close resemblance to Tylopus jeekeli Golovatch & Enghoff, 1993.

##### Diagnosis:

Very similar to Tylopus jeekeli, especially as regards its gonopod conformation, but differs in the paraterga lying much lower (at ca 1/3 versus 1/4–1/5 midbody height), in the caudal corners of the paraterga protruding behind the rear tergal contour already from segment 16 (versus segment 2), and also in gonopod spine **z** being much smaller and placed closer to the base of spine **h**.

##### Description:

Length 31 mm (holotype) or 31.5 mm (♂), width of midbody pro- and metazona 2.4 and 3.2 mm (holotype) or 2.3 and 3.4 mm (♂), respectively. Coloration of live animals and alcohol material uniformly blackish-brown (Fig. 13A–G); calluses of paraterga a little lighter, brown; antennomeres 1–5 light brown to yellowish, legs and venter light brown to grey-yellowish (Fig. 13A–G).

All characters as in Tylopus bispinosus sp. n., except as follows.

Antennae rather short and slender, reaching behind segment 3 dorsally. In width, head = segment 3 < collum < segments 2 and 4 < 5–16; thereafter body gradually and gently tapering towards telson (Fig. 1B).

Paraterga on collum like large rounded flaps (Fig. 13B, C). Following paraterga lying at about 1/3 midbody height, evidently declined ventrolaterally, subhorizontal only on a few posteriormost segments, mostly pointed caudally, subrectangular until segment 15, thereafter increasingly well protruding behind rear tergal contour (Fig. 13A–G). Metaterga with 2(3)+2(3) and 3–5+3–5 long setae arranged in two transverse rows. Axial line present on both halves of metaterga. Transverse sulcus present on segments 5–18, very finely beaded at bottom (Fig. 13B, F, G). Stricture between pro- and metazona finely striolate (Fig. 13B, D). Epiproct tip broad and emarginate (Fig. 13G, H). Hypoproct semi-circular, both caudal setae widely separated (Fig. 13H). Pleurosternal carinae as complete ridges on segments 2–4, thereafter retained until segment 17 mostly as a small caudal tooth (Fig. 13C–F).

A low, only slightly divided, setose lobe between coxae 4 (Fig. 13I, J). Legs relatively short, ca 1.6–1.7 times as long as midbody height (Fig. 13K). Femora evidently bulged laterally (Fig. 13K); all postgonopodal legs except two last pairs with an evident adenostyle in parabasal 1/3 of each postfemur and tibia; tarsal brushes missing; all telopoditomeres except tarsi with dense adpressed pilosity (Fig. 13K).

**Figure 14. F14:**
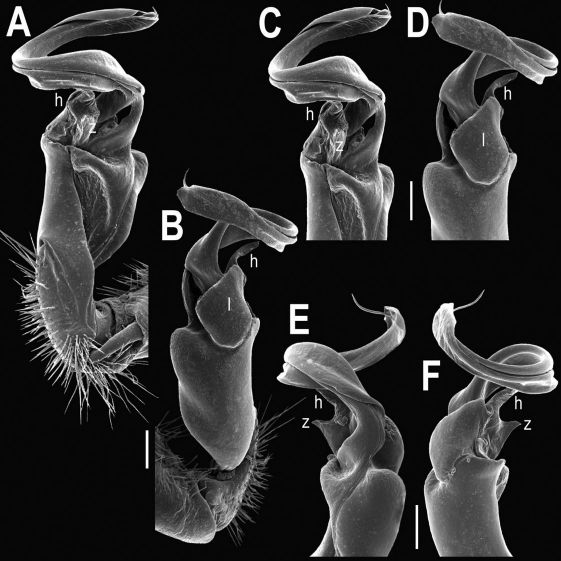
Tylopus parajeekeli sp. n., ♂ paratype. **A, B** right gonopod, mesal and lateral views, respectively **C–F** distal part of right gonopod, submesal, sublateral, subcaudal and suboral views, respectively. Scale bar: 0.2 mm.

**Figure 15.**
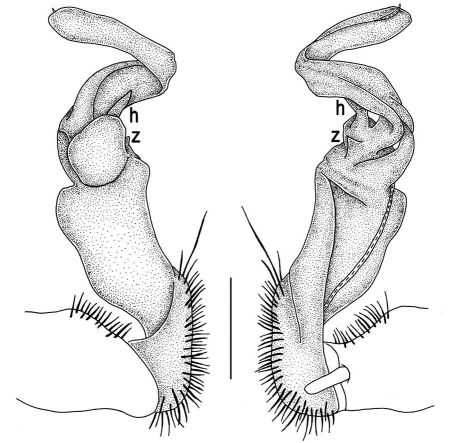
Tylopus parajeekeli sp. n., ♂ paratype. **A, B** right gonopod, lateral and mesal views, respectively. Scale bar: 0.5 mm.

Gonopods (Figs 14, 15) with lobe **l** well-demarcated; spine **h** small, but elongate, not bifid; spine **z** very small, dentiform, placed at base of spine **h**.

### New faunistic records

The following seven species have been illustrated in additional detail to confirm their identities, as well as to provide further information concerning both their variation and distribution.

#### Tylopus allorugosus

Golovatch & Enghoff, 1993

**Figure 16.**
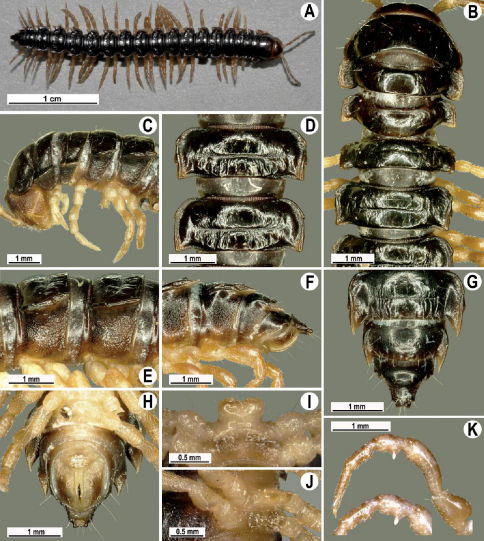
Tylopus allorugosus Golovatch & Enghoff, 1993, ♂ from 10 km before Doi Inthanon summit (**A–K**). **A** habitus, live coloration **B, C** anterior part of body, dorsal and lateral views, respectively **D, E** segments 10 and 11, dorsal and lateral views, respectively **F, G, H** posterior part of body, lateral, dorsal and ventral views, respectively **I, J** sternal cones between coxae 4, subcaudal and sublateral views, respectively **K** midbody leg.

Figs 16 [Fig F17] 18

Tylopus allorugosus Golovatch and Enghoff 1993: 100.
Tylopus allorugosus : Enghoff 2005: 98.

##### Material:

2 ♂ (CUMZ), Thailand, Chiang Mai Province, Chom Thong District, Doi Inthanon National Park, Siriphum Waterfall, ca 1320 m, 18°32'49N, 98°30'57E, 13.10.2009, leg. S. Panha, J. Sutcharit & N. Likhitrakarn; 2 ♂ (CUMZ), same locality, main road, 10 km before summit, ca 1700 m, 18°31'15N, 98°30'1E, 13.10.2009, leg. S. Panha, J. Sutcharit & N. Likhitrakarn.

**Figure 17. F17:**
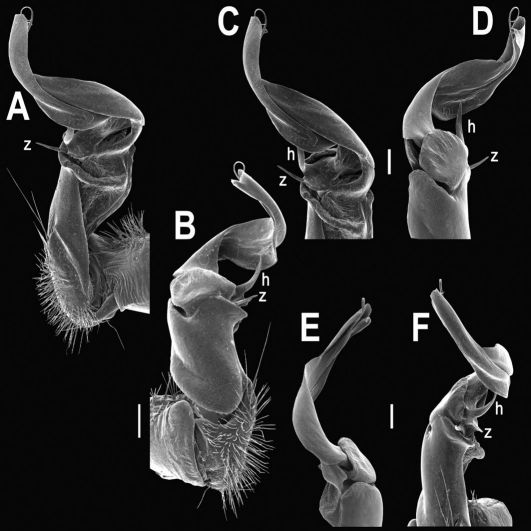
Tylopus allorugosus Golovatch & Enghoff, 1993, ♂ from 10 km before Doi Inthanon summit. **A, B** right gonopod, mesal and lateral views, respectively **C–F** distal part of right gonopod, submesal, sublateral, subcaudal and suboral views, respectively. Scale bar: 0.2 mm.

**Figure 18.**
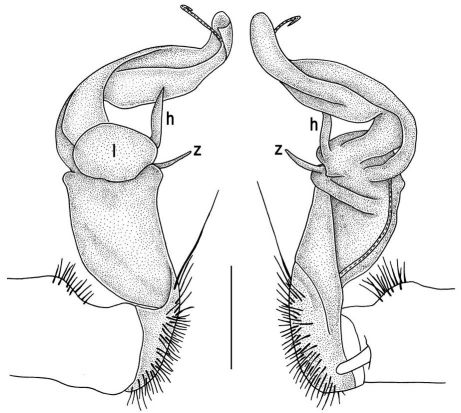
Tylopus allorugosus Golovatch & Enghoff, 1993, ♂ from 10 km before Doi Inthanon summit. **A, B** right gonopod, lateral and mesal views, respectively. Scale bar: 0.5 mm.

##### Remarks.

This strictly topotypic material fully agrees with the original description (Golovatch and Enghoff 1993), showing no evident variation in peripheral and gonopod structure (Figs 16–18).

#### Tylopus degerboelae

Golovatch & Enghoff, 1993

**Figure 19.**
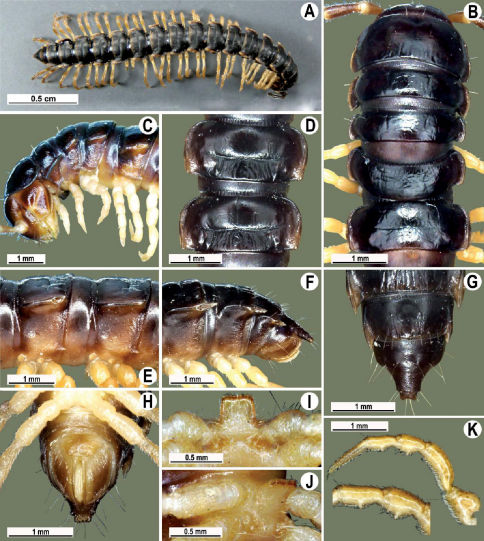
Tylopus degerboelae Golovatch & Enghoff, 1993, ♂ from Doi Suthep National Park (**A–K**). **A** habitus, live coloration **B, C** anterior part of body, dorsal and lateral views, respectively **D, E** segments 10 and 11, dorsal and lateral views, respectively **F, G, H** posterior part of body, lateral, dorsal and ventral views, respectively **I, J** sternal cones between coxae 4, subcaudal and sublateral views, respectively **K** midbody leg.

Figs 19 [Fig F20] 21

Tylopus degerboelae Golovatch and Enghoff 1993: 111.
Tylopus degerboelae :Enghoff 2005: 99.

##### Material:

3 ♂ (CUMZ), Thailand, Chiang Mai Province, Mueang Chiang Mai District, Doi Suthep National Park, ca 1300 m, 18°48'9N, 98°54'11E, 20.04.2009, leg. S. Panha, J. Sutcharit & N. Likhitrakarn; 4 ♂, 3 ♀, 2 juv. (CUMZ), same Province, WiangKaen District, Doi Phatang, 6.07.2009, leg. S. Panha & J. Sutcharit.

**Figure 20. F20:**
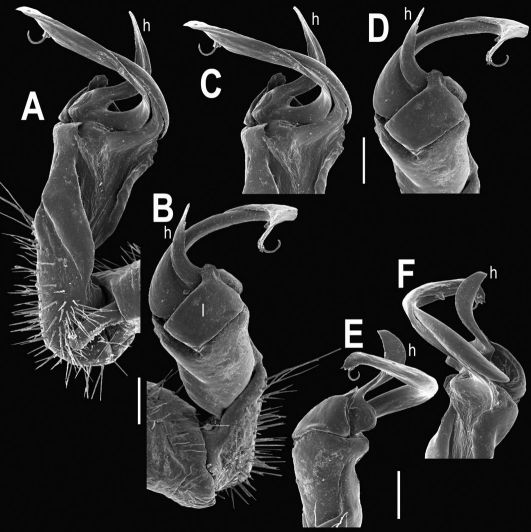
Tylopus degerboelae Golovatch & Enghoff, 1993, ♂ from Doi Suthep National Park. **A, B** right gonopod, mesal and lateral views, respectively **C–F** distal part of right gonopod, mesal, lateral, subcaudal and suboral views, respectively. Scale bar: 0.2 mm.

**Figure 21.**
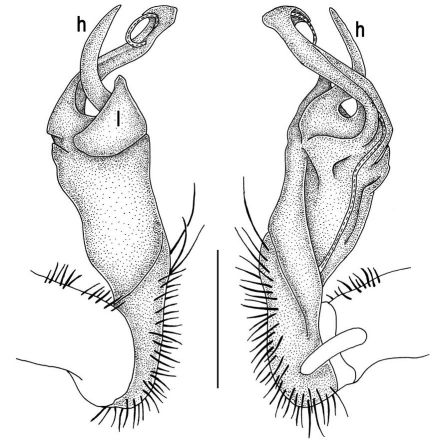
Tylopus degerboelae Golovatch & Enghoff, 1993, ♂ from Doi Suthep National Park. **A, B** right gonopod, lateral and mesal views, respectively. Scale bar: 0.5 mm.

##### Remarks.

This partly topotypic material fully agrees with the original description (Golovatch and Enghoff 1993), showing only slight variation in general coloration (ranging from pale castaneous to piceous), in ♂ prefemora often being considerably bulged laterally, and in the tip of lobe **l** of the gonopod often devoid of apical denticles (Figs 19–21).

#### Tylopus haplorugosus

Golovatch & Enghoff, 1993

**Figure 22.**
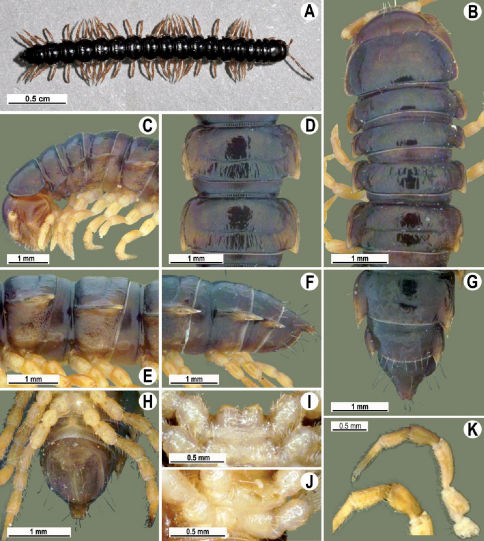
Tylopus haplorugosus Golovatch & Enghoff, 1993, ♂ (**A–K**). **A** habitus, live coloration **B, C** anterior part of body, dorsal and lateral views, respectively **D, E** segments 10 and 11, dorsal and lateral views, respectively **F, G, H** posterior part of body, lateral, dorsal and ventral views, respectively **I, J** sternal cones between coxae 4, subcaudal and sublateral views, respectively **K** midbody leg.

Figs 22 [Fig F23] 24

Tylopus haplorugosus Golovatch and Enghoff 1993: 99.
Tylopus haplorugosus : Enghoff 2005: 99.

##### Material:

1 ♂ (CUMZ), Thailand, Chiang Mai Province, Chom Thong District, Doi Inthanon National Park, main road, 10 km before summit, ca 1700 m, 18°31'15N, 98°30'1E, 13.10.2009, leg. S. Panha, J. Sutcharit & N. Likhitrakarn.

**Figure 23. F23:**
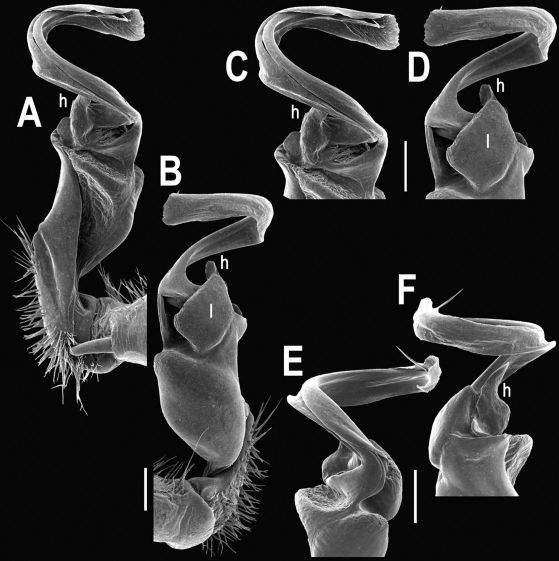
Tylopus haplorugosus Golovatch & Enghoff, 1993, ♂. **A, B** right gonopod, mesal and lateral views, respectively **C–F** distal part of right gonopod, mesal, lateral, subcaudal and suboral views, respectively. Scale bar: 0.2 mm.

**Figure 24.**
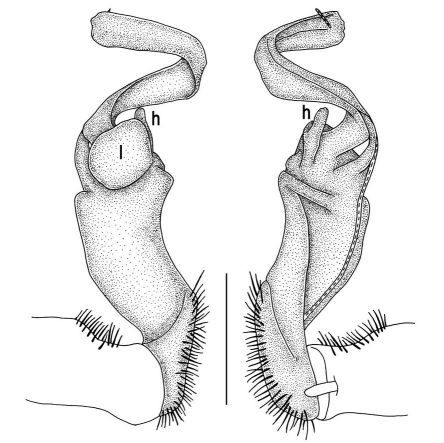
Tylopus haplorugosus Golovatch & Enghoff, 1993, ♂. **A, B** right gonopod, lateral and mesal views, respectively. Scale bar: 0.5 mm.

##### Remarks.

This strictly topotypic material fully agrees with the original description (Golovatch and Enghoff 1993), showing no evident variation in peripheral and gonopod structure (Figs 22–24).

#### Tylopus jeekeli

Golovatch & Enghoff, 1993

**Figure 25.**
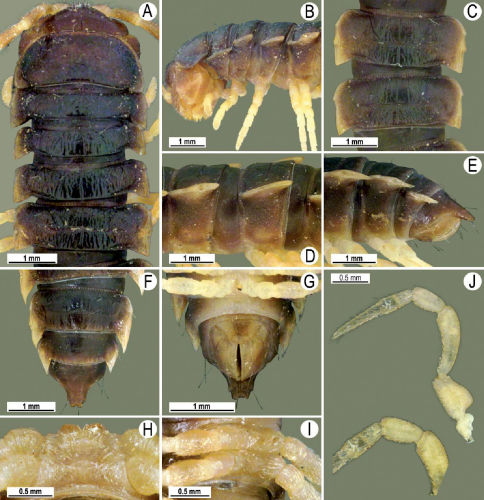
Tylopus jeekeli Golovatch & Enghoff, 1993, ♂ (**A–J**). **A, B** anterior part of body, dorsal and lateral views, respectively. **C, D** segments 10 and 11, dorsal and lateral views, respectively. **E, F, G** posterior part of body, lateral, dorsal and ventral views, respectively **H, I** sternal cones between coxae 4, subcaudal and sublateral views, respectively **J** midbody leg.

Figs 25 [Fig F26] 27

Tylopus jeekeli Golovatch and Enghoff 1993: 108.
Tylopus jeekeli : Enghoff 2005: 99.

##### Material:

4 ♂, 7 ♀, 1 juv. (CUMZ), Thailand, Chiang Mai Province, Mueang Chiang Mai District, Doi Suthep National Park, ca 1300 m, 18°48'9N, 98°54'11E, 22.10.2009, leg. S. Panha, J. Sutcharit & N. Likhitrakarn.

**Figure 26. F26:**
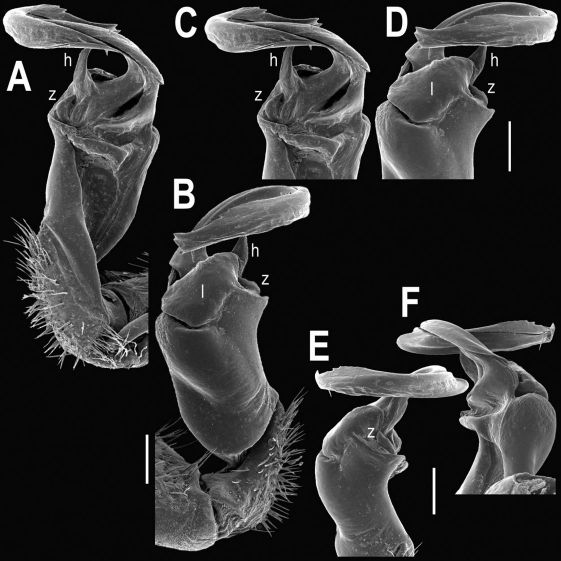
Tylopus jeekeli Golovatch & Enghoff, 1993, ♂. **A, B** right gonopod, mesal and lateral views, respectively **C–F** distal part of right gonopod, mesal, lateral, suboral and subcaudal views, respectively. Scale bar: 0.2 mm.

**Figure 27.**
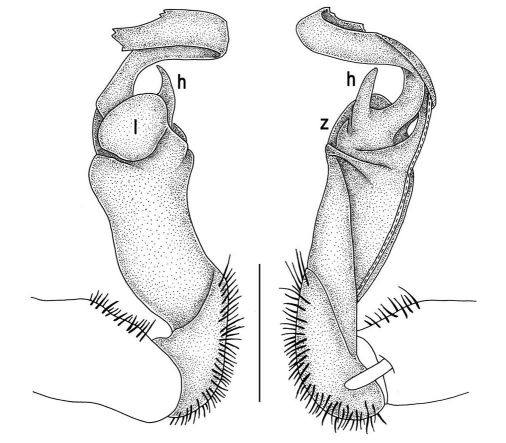
Tylopus jeekeli Golovatch & Enghoff, 1993, ♂. **A, B** right gonopod, lateral and mesal views, respectively. Scale bar: 0.5 mm.

##### Remarks.

This represents a second record of this species, the type locality being Doi Inthanon National Park in the same province. Our material almost fully agrees with the original description (Golovatch and Enghoff 1993), showing slight variation only in spine **h** of the gonopod being non-bifid, but simple and entire (Figs 25–27).

#### Tylopus prosperus

Golovatch & Enghoff, 1993

**Figure 28.**
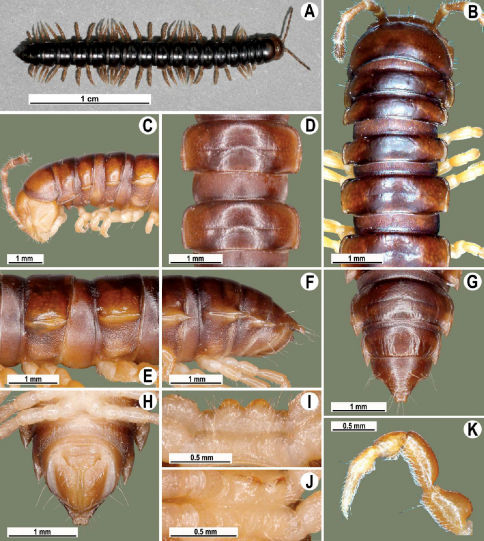
Tylopus prosperus Golovatch & Enghoff, 1993, ♂ (**A–K**). **A** habitus, live coloration **B, C** anterior part of body, dorsal and lateral views, respectively **D, E** segments 10 and 11, dorsal and lateral views, respectively **F, G, H** posterior part of body, lateral, dorsal and ventral views, respectively **I, J** sternal cones between coxae 4, subcaudal and sublateral views, respectively **K** midbody leg.

Figs 28 [Fig F29] 30

Tylopus prosperus Golovatch and Enghoff 1993: 93.
Tylopus prosperus : Enghoff 2005: 99.

##### Material:

2 ♂ (CUMZ), Thailand, Chiang Mai Province, Chom Thong District, Doi Inthanon National Park, summit, 2520 m, 18°34'29N, 98°28'48E, 12.10.2009, leg. S. Panha, J. Sutcharit & N. Likhitrakarn.

**Figure 29. F29:**
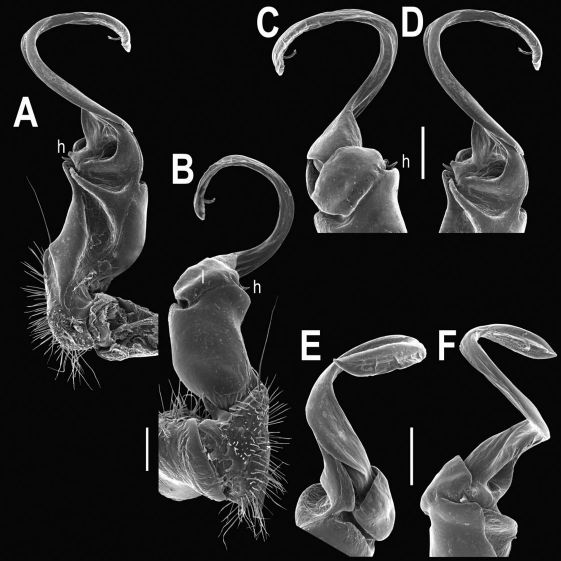
Tylopus prosperus Golovatch & Enghoff, 1993, ♂. **A, B** right gonopod, mesal and lateral views, respectively **C–F** distal part of right gonopod, sublateral, mesal, suboral and subcaudal views, respectively. Scale bar: 0.2 mm.

**Figure 30.**
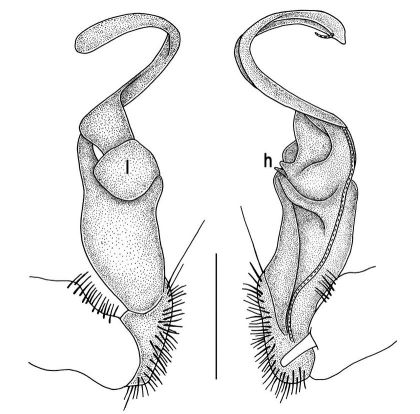
Tylopus prosperus Golovatch & Enghoff, 1993, ♂. **A, B** right gonopod, lateral and mesal views, respectively. Scale bar: 0.5 mm.

##### Remarks.

This strictly topotypic material fully agrees with the original description (Golovatch and Enghoff 1993), showing no evident variation in peripheral and gonopod structure (Figs 28–30).

#### Tylopus rugosus

Golovatch & Enghoff, 1993

**Figure 31.**
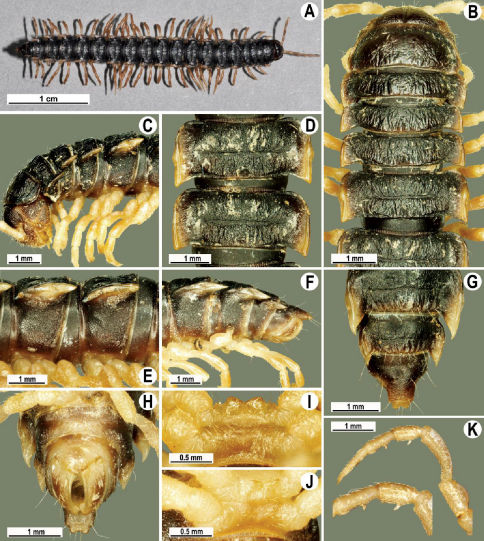
Tylopus rugosus Golovatch & Enghoff, 1993, ♂ (**A–K**). **A** habitus, live coloration **B, C** anterior part of body, dorsal and lateral views, respectively **D, E** segments 10 and 11, dorsal and lateral views, respectively **F, G, H** posterior part of body, lateral, dorsal and ventral views, respectively **I, J** sternal cones between coxae 4, subcaudal and sublateral views, respectively **K** midbody leg.

Figs 31 [Fig F32] 33

Tylopus rugosus Golovatch and Enghoff 1993: 95.
Tylopus rugosus : Enghoff 2005: 99.

##### Material:

4 ♂ (CUMZ), Thailand, Chiang Mai Province, Phrao District, Buathong Waterfall forest park, 510 m, 19°4'10N, 99°4'46E, 29.09.2009, leg. N. Likhitrakarn.

**Figure 32. F32:**
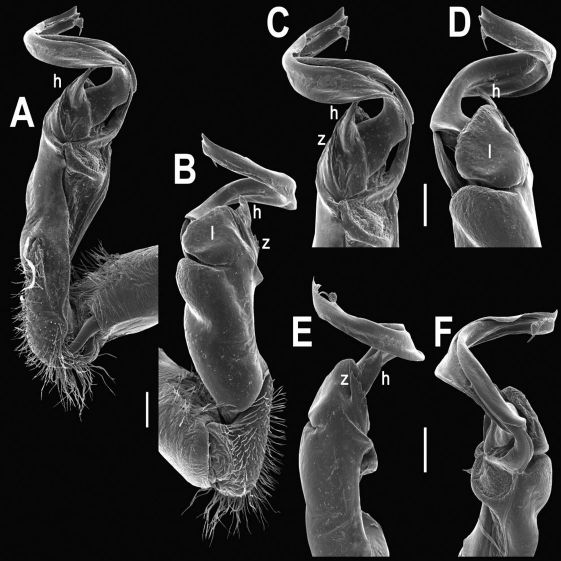
Tylopus rugosus Golovatch & Enghoff, 1993, ♂. **A, B** right gonopod, mesal and lateral views, respectively **C–F** distal part of right gonopod, mesal, lateral, suboral and subcaudal views, respectively. Scale bar: 0.2 mm.

**Figure 33.**
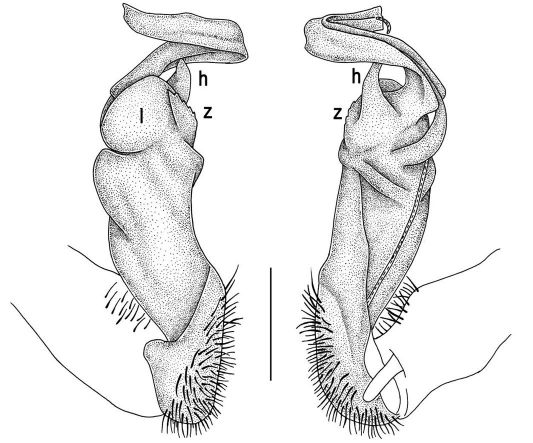
Tylopus rugosus Golovatch & Enghoff, 1993, ♂. **A, B** right gonopod, lateral and mesal views, respectively. Scale bar: 0.5 mm.

##### Remarks.

This near-topotypic material fully agrees with the original description (Golovatch and Enghoff 1993), showing no evident variation in peripheral and gonopod structure (Figs 31–33).

#### Tylopus perarmatus

Hoffman, 1973

**Figure 34.**
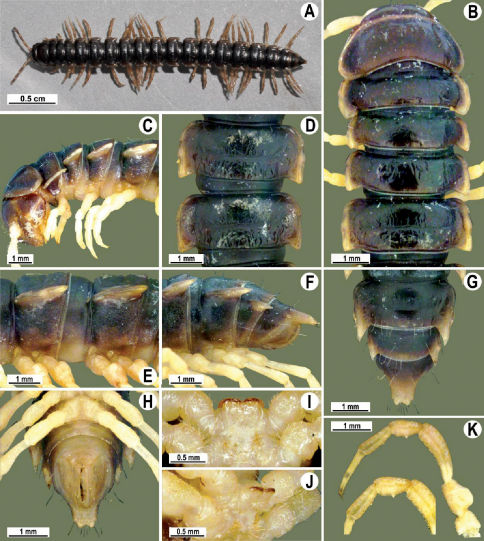
Tylopus perarmatus Hoffman, 1973, ♂ from Ton Tong Waterfall (**A–K**). **A** habitus, live coloration **B, C** anterior part of body, dorsal and lateral views, respectively **D, E** segments 10 and 11, dorsal and lateral views, respectively **F, G, H** posterior part of body, lateral, dorsal and ventral views, respectively **I, J** sternal cones between coxae 4, subcaudal and sublateral views, respectively **K** midbody leg.

Figs 34 [Fig F35] [Fig F36] [Fig F37] 38

Tylopus perarmatus Hoffman 1973: 372.
Tylopus perarmatus : Golovatch and Enghoff 1993: 106.
Tylopus perarmatus : Enghoff 2005: 99.

##### Material:

5 ♂, 3 ♀, 1 juv. (CUMZ), Thailand, Chiang Mai Province, Chom Thong District, Doi Inthanon National Park, Siriphum Waterfall, ca 1320 m, 18°32'49N, 98°30'57E, 13.10.2009, leg. S. Panha, J. Sutcharit & N. Likhitrakarn; 2 ♂, 2 ♀ (CUMZ), same province, Wiang Kaen District, Doi Phatang, 25.10.2008, leg. S. Panha & J. Sutcharit; 5 ♂, 2 ♀ (CUMZ), Lampang Province, Ngao District, Thum Pha Thai, 23.10.2008, leg. S. Panha, J. Sutcharit & N. Likhitrakarn; 1 ♂, 1 ♀ (CUMZ), Chiang Rai Province, Mueang Chiang Rai District, Ban Pang Rim Kon, 10.07.2006, leg. S. Panha; 1 ♂ (CUMZ), same province, Thoeng District, Phucheefah, 10.07.2006, leg. S. Panha & J. Sutcharit; 2 ♂, 1 ♀ (CUMZ), same province, Wiang Kaen District, Doi Pha Tang, 10.07.2006; 1 ♂, 2 ♀ (CUMZ), Phayao Province, Chiang Kham District, Nam Min Waterfall, 23.10.2008, leg. S. Panha & J. Sutcharit; 2 ♂, 2 ♀ (CUMZ), Phrae Province, Rong Kwang District, Tham Pha Nang Khoi, ca 280 m, 18°22'10N, 100°21'12E, 9.10.2009, leg. S. Panha, J. Sutcharit & N. Likhitrakarn; 2 ♂, 2 ♀ (CUMZ), same locality, 29.09.2010, leg. J. Sutcharit & P. Pimvichai; 9 ♂, 6 ♀, 2 juv. (CUMZ), Nan Province, Pua District, Ton Tong Waterfall, ca 1130 m, 19°12'36N, 101°4'14E, 10.10.2009, leg. S. Panha, J. Sutcharit & N. Likhitrakarn.

**Figure 35. F35:**
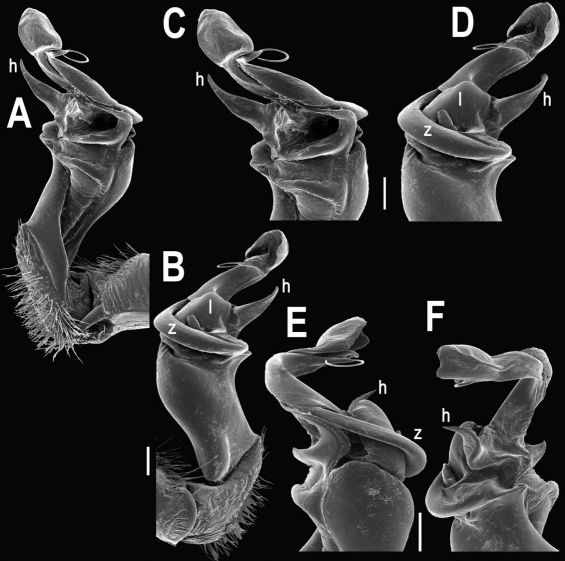
Tylopus perarmatus Hoffman, 1973, ♂ from Ton Tong Waterfall. **A, B** right gonopod, mesal and lateral views, respectively **C–F** distal part of right gonopod, mesal, lateral, suboral and subcaudal views, respectively. Scale bar: 0.2 mm.

**Figure 36. F36:**
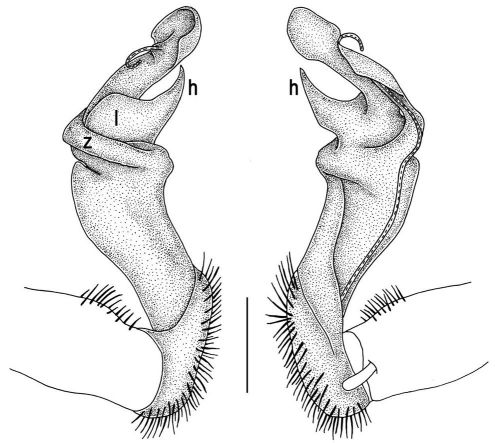
Tylopus perarmatus Hoffman, 1973, ♂ from Ton Tong Waterfall. **A, B** right gonopod, lateral and mesal views, respectively. Scale bar: 0.5 mm.

**Figure 37. F37:**
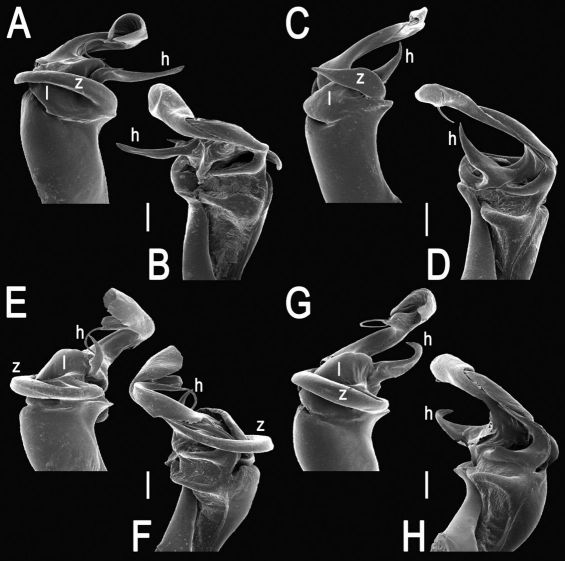
Tylopus perarmatus Hoffman, 1973, ♂ from Ban Pang Rim Kon (**A, B**), ♂ from Ton Tong Waterfall (**C, D**), ♂ from Phucheefah (**E, F**), and ♂ from Ton Tong Waterfall (**G, H**). **A–H** distal part of right gonopod, lateral, mesal, lateral, mesal, lateral, mesal, lateral, and mesal views, respectively. Scale bar: 0.2 mm.

**Figure 38.**
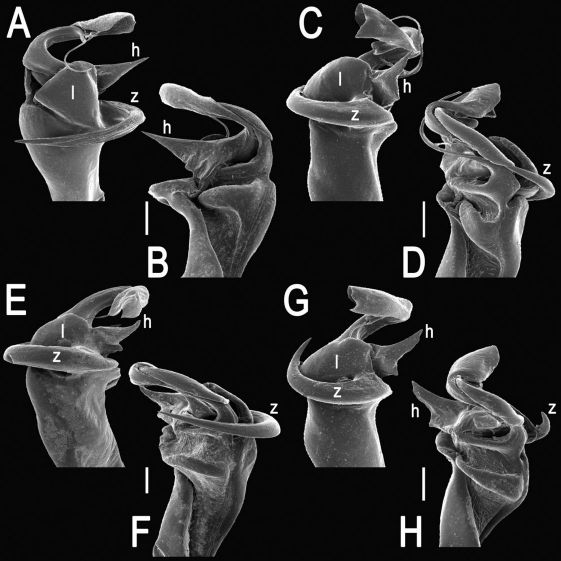
Tylopus perarmatus Hoffman, 1973, ♂ from Siriphum Waterfall (**A, B**), ♂ from Thum Pha Thai (**C, D**), ♂ from Tham Pha Nang Khoi (**E, F**), and ♂ from Thum Pha Thai (**G, H**). **A–H**: distal part of right gonopod, lateral, mesal, lateral, mesal, lateral, mesal, lateral, and mesal views, respectively. Scale bar: 0.2 mm.

##### Remarks.

This species has long been known as perhaps the most widespread and common congener in northern Thailand, also showing considerable variation both in body texture and gonopod structure (Golovatch and Enghoff 1993). The new samples add to this variation in the gonopods often with spine **h** rather narrow and spiniform to broadly denti- or lobiform, and spine **z** nearly straight to strongly unciform (Figs 34–38).

### A key to species of Tylopus (based chiefly on ♂)

**Table:** 

1	Most ♂ prefemora evidently swollen laterally (Figs 1K, 4J, 7K, 10K, 13K, 16K, 19K, 22K, 25K, 28K, 31K, 34K)	2
–	All ♂ prefemora normal, not bulged laterally	25
2	Surface of metaterga virtually smooth, at best extremely faintly rugulose in certain places and/or with a few barely traceable (setiferous) tubercles near caudal margin (setae mostly broken off)	3
–	Surface of metaterga mostly rugulose to coarsely rugose/tuberculate	6
3	Paraterga moderately developed (Fig. 28A–G), ratio of ♂ midbody prozonite to metazonite width ca 1:1.15. Transverse sulcus on metaterga starting from segment 5, but fully developed and reaching base of paraterga only from segment 6. Calluses without incisions (Fig. 28A–G). Gonopod solenophore particularly slender (Figs 29, 30)	Tylopus prosperus
–	Paraterga relatively well-developed, radio of ♂ midbody prozonite to metazonite width over 1:1.2. Transverse sulcus on metaterga starting from segment 4 or 5, always fully developed and reaching base of paraterga on segment 5	4
4	Calluses without incisions. Gonopod postfemoral lobe **l** much broader than long; area basal to **l** delimited by a distinct cingulum	Tylopus magicus
–	Calluses mostly with 1–2 incisions. Gonopod postfemoral lobe **l** either as long as broad or longer; no cingulum basal to **l**	5
5	Metatergal surface entirely smooth, polished, without tubercles. Midline wanting. Pleurosternal carinae relatively weak, as small teeth only on a few anteriormost segments; ♂ legs without adenostyles (= tubercles). Gonopods with three rather small, spiniform processes near base of lobe **l**	Tylopus mutilatus
–	Metaterga at best only very faintly rugulose near waist, near sulcus and/or at base of paraterga, with 2–3 weak, oblong tubercles near rear margin. Midline mostly traceable at least on anterior halves of metaterga. Pleurosternal carinae more strongly developed; most ♂ postfemora and tibiae tuberculiferous. Gonopods with only two larger outgrowths near base of lobe **l**	Tylopus similirugosus
6	Metaterga without evident setiferous tubercles, only sometimes with very small, rudimentary wrinkles or knobs	7
–	Metaterga with evident setiferous tubercles	11
7	Body larger: 38–42 mm long, 2.8–3.8 and 4.3–5.0 mm wide on pro- and metaterga, respectively. Gonopod with a short spiniform process **h**, a basally only poorly delimited lobe **l**, and a small lobiform process **z** (Figs 5, 6)	Tylopus grandis sp. n.
–	Body smaller. Gonopod otherwise	8
8	Both processes **h** and **z** of the gonopod spiniform (Figs 2–3)	Tylopus bispinosus sp. n.
–	Gonopod otherwise	9
9	Gonopod process **h** subflagelliform, process **m** extremely long and prominent (Figs 8, 9)	Tylopus extremus sp. n.
–	Gonopod otherwise	10
10	♂ legs shorter, ca 1.2–1.3 times as long as midbody height (Fig. 10K). Gonopod lobe **l** velum-shaped and supplied with two denticles; spine **z** short and knife-shaped while spine **h** rudimentary (Figs 11, 12)	Tylopus veliger sp. n
–	♂ legs longer, ca 1.6–1.7 times as long as midbody height (Fig. 13K). Gonopod spine **z** small, placed closer to base of spine **h** (Figs 14, 15)	Tylopus parajeekeli sp. n.
11	Most metaterga with a pattern of 2+2 and 2+2 setiferous tubercles in two rows, rear row somewhat less strongly developed than fore one	Tylopus doriae
–	Most metaterga with rear row of setiferous tubercles or wrinkles more strongly developed than fore row, the latter (next to) wanting	12
12	Transverse sulcus on metaterga starting from segment 4, either fully or almost fully developed there, always fully developed from segment 5	13
–	Transverse sulcus on metaterga starting only from segment 5	16
13	Transverse sulcus fully developed and reaching base of paraterga already from segment 4. Gonopod tooth **z** at base of lobe **l** coarsely serrate along proximal margin	Tylopus hilaris
–	Transverse sulcus fully developed only from segment 5. Gonopod tooth **z** either devoid of serration or serrate along distal margin	14
14	Paraterga 2 caudolaterally rather broadly rounded. Gonopod relatively simple, process **h** poorly developed, no additional outgrowths near base	Tylopus affinis
–	Paraterga 2 caudally pointed. Gonopods more complex	15
15	Coloration dark brown, without cingulate pattern. Sternal lamina between ♂ coxae 4 low and distinctly bimodal (Fig. 31I, J). Gonopods (Figs 32, 33) with tooth **z** prominent and serrate along distal margin	Tylopus rugosus
–	Coloration pale, with a cingulate pattern. Sternal lamina between ♂ coxae 4 high, subquadrate. Gonopod tooth **z** smaller and spiniform	Tylopus semirugosus
16	Paratergal corner protruding caudad beyond rear contour only from segment 15, being obtusangular or subrectangular and lying more or less within the contour until segment 14	Tylopus hilaroides
–	Paratergal corner protruding caudad before segment 14, mostly pointed	17
17	Pattern of tergal setation on segments 18 and/or 19: 2+2 and 5+5 in two rows	18
–	Pattern of tergal setation at least on segments 5–19: 2+2 and 4+4 in two rows	21
18	Pattern of tergal setation 2+2 and 5+5 on both segments 18 and 19. Paraterga 2 caudally pointed. Epiproct with pre-apical incisions very close to apical knobs. Sternal lamina between ♂ coxae 4 an unusually low, even ridge. Adenostyles on midbody ♂ postfemora and, to a lesser extent, tibiae exceptionally prominent	Tylopus poolpermorum
–	Pattern of tergal setation 2+2 and 5+5 on segment 19. Paraterga 2 more or less narrowly rounded. Pre-apical incisions on epiproct better removed from tip. Sternal lamina between ♂ coxae 4 concave medially. Ventral adenostyles on ♂ legs less prominent	19
19	Body smaller: width ca 2.0 mm. Sternal lamina between ♂ coxae 4 as a pair of separate, setiferous tubercles (Fig. 22I, J). Ventral adenostyles on ♂ legs almost missing (Fig. 22K). Gonopods without any outgrowth near base of process **h** (Figs 23, 24)	Tylopus haplorugosus
–	Body larger: width over 3.0 mm. Sternal lamina between ♂ coxae 4 single. Ventral adenostyles on ♂ legs more prominent. Gonopod with a spine near base of process **h**	20
20	Sternal lamina between ♂ coxae 4 high, emarginate (Fig. 16I, J). Adenostyles on ♂ postfemora and tibiae well-developed (Fig. 16K). Gonopods rather simple, spine **z** inconspicuous (Figs 17, 18)	Tylopus allorugosus
–	Sternal lamina between ♂ coxae lower, slightly concave. Adenostyles on ♂ postfemora and tibiae less strongly developed. Gonopods more complex, spine **z** long and large (Figs 35–38)	Tylopus perarmatus
21	Paraterga 2 pointed caudally. Sternal lamina between ♂ coxae 4 exceptionally densely setose, low, concave ventrally (Fig. 25I, J). Gonopods with a medium-sized process **h**, and a smaller lobular **z** at base of **h** (Figs 26, 27)	Tylopus jeekeli
–	Paraterga 2 more or less narrowly rounded caudally. Sternal lamina between ♂ coxae 4 higher and less strongly setose. Gonopod outgrowths **h** and **z** either almost wanting or very large	22
22	Sternal lamina between ♂ coxae 4 with a straight ventral margin. Pleurosternal carinae poorly developed, in ♂ slightly projecting caudad beyond rear margin only until segments 8–10	23
–	Sternal lamina between ♂ coxae 4 slightly concave ventrally. Pleurosternal carinae better developed, in ♂ slightly projecting caudad beyond rear margin at least till segment 15	24
23	Body smaller: width up to 3.1–3.2 mm. Mid-dorsal line very clear on both halves of metaterga. Gonopods relatively simple, with both **h** and **z** almost wanting	Tylopus hoffmani
–	Body larger: width 4.0–5.3 mm. Mid-dorsal line not so well-developed at least on rear halves of metaterga. Gonopods more complex, with both **h** and **z** very conspicuous	Tylopus baenzigeri
24	Metatergum 19 slightly rugulose posteriorly. Calluses on segment 2 with three, on following paraterga with two, incisions. Gonopods extremely complex, with numerous spiniform outgrowths	Tylopus perplexus
–	Metatergum 19 entirely smooth. Calluses with two and three incisions on poreless and poriferous paraterga, respectively. Gonopod less strongly differentiated	Tylopus amicus
25	Either most of ♂ sterna with oblique tubercles or spines, or only anterior sterna with small cones near coxae	26
–	Neither spines nor tubercles on ♂ sterna	28
26	Only rear sternum on most of ♂ segments with a pair of small spines. Metaterga mostly with 2+2 and 3+3 setiferous tubercles in two transverse rows. Gonopod process **h** and lobe **l** relatively well-developed	Tylopus silvestris
–	Fore and rear sterna of most of ♂ segments with a pair of tubercles and spines, respectively. Fore row of tergal setae not borne on tubercles, rear row on 2+2 tubercles	27
27	Transverse sulcus starting from metatergum 4, fully developed from metatergum 5. ♂ tarsal brushes missing. Northern Vietnam	Tylopus maculatus
–	Transverse sulcus starting from metatergum 3, still underdeveloped on metatergum 4, fully developed from metatergum 5. ♂ tarsal brushes present only on a few anteriormost legs. Yunnan, China	Tylopus sinensis
28	Metaterga entirely smooth and polished, devoid of evident tubercles, at best extremely faintly rugulose near transverse sulcus	29
–	Metaterga rather clearly rugose/tuberculate/granulate, posterior row of setae at least partly borne on tubercles	32
29	Transverse sulcus on metaterga starting from segment 4, but fully developed and reaching base of paraterga only from segment 5. Ventral adenostyles on ♂ legs: a distal knob on femur, a distomedial knob on postfemur, and a parabasal knob on both tibia and tarsus	30
–	Transverse sulcus on metaterga starting only from segment 5. Pattern of ♂ leg adenostyles otherwise	31
30	Head a little wider than collum and subequal in width to segment 3. Paraterga caudally considerably acutangular and beak-shaped only from segment 14. Gonopod process **h** at about midlength with a strong ventral outgrowth	Tylopus procurvus
–	Head a little narrower than collum and subequal in width to segment 2. Paraterga caudally beak-shaped already from segment 7, especially strongly so from segment 12. Gonopod process **h** without outgrowth	Tylopus crassipes
31	Larger species: body width 3.1 mm. Pattern of tergal setation: 2+2 and 3+3 to 6+6 in two rows, rear row easily traceable due to insertion points. Metaterga very finely rugulose only near transverse sulcus. Epiproct unusually broad. Pads instead of adenostyles on ♂ femora, postfemora, tibiae (all distally) and tarsi (almost entirely)	Tylopus pulvinipes
–	Small species: width 1.6 mm. Only a single row of 2+2 tergal setae. Metaterga entirely smooth. Epiproct not so wide. Adenostyles on ♂ legs present	Tylopus sigma
32	Metatergal surface polished and smooth except for conspicuous tubercles in two rows	33
–	Metaterga at least partly rugulose/rugose to granular; at most one row of tubercles	35
33	Paraterga very poorly developed, rounded, low, projecting slightly caudad beyond rear contour like small knobs only on segments 18 and 19. Calluses virtually devoid of incisions. Transverse sulcus on metaterga poorly developed, starting already from segment 2, although fully developed only from segment 5. A paramedian pair of denticles between ♂ coxae 5 behind a prominent, subquadrate lamina between ♂ coxae 4. Gonopod process **h** entirely missing, lobe **l** normal	Tylopus strongylosomoides
–	Paraterga better developed, protruding caudad beyond rear contour at least from segment 5. Calluses always at least with one lateral incision. Transverse sulcus starting only from segments 3–5. Dentiform tubercles between ♂ coxae 5 missing. Gonopod process **h** invariably present, lobe **l** with a spine apically	34
34	Paraterga acutangular caudally and pointed beak-like already from collum. Tergal setiferous tubercles: 3+3 and 5+5 on segments 16–19. Adenostyle pattern on ♂ legs: a distal knob on femora and a parabasal knob on most of postfemora, tibiae, and tarsi. Gonopod process **h** large, lamellar, sigmoid	Tylopus granulatus
–	Paraterga acutangular caudally and pointed beak-like only from segment 4. 2+2 and 4+4 tergal setiferous tubercles on segments 16–19. Adenostyle pattern on ♂ legs: a proximal finger-shaped tubercle crowned with a bunch of setae only on femora 6, 8, and 9. Gonopod process **h** smaller, spiniform	Tylopus topali
35	Transverse sulcus on metaterga starting and fully developed from segment 5. Ventral adenostyles present on all ♂ podomeres except coxa	36
–	Transverse sulcus on metaterga starting from segment 4, but fully developed only from segment 5. Ventral tubercles only on some of ♂ telopoditomeres	37
36	Metaterga rugulose also in front of transverse sulcus, at rear margin with several oblong tubercles. Sternal lamina between ♂ coxae 4 like a pair of setiferous knobs. Neither gonopod lobe **m** nor lobe **l** spinigerous	Tylopus tamdaoensis
–	Metaterga rugose only behind transverse sulcus, without evident turbercles at rear margin. Sternal lamina between ♂ coxae 4 single. Both gonopod lobe **m** and lobe **l** crowned with a spine	Tylopus nodulipes
37	Metaterga modestly rugulose only near transverse sulcus, posteriorly neither granular nor microtuberculate. Calluses broad. Gonopod process **h** simple, high, never particularly coiled; lobe **l** very modestly serrate at apex	38
–	Metaterga distinctly rugose-granular/microtuberculate even on fore halves. Calluses narrow. Gonopod process **h** better developed and more strongly coiled; lobe **l** apically either bare or with a digitiform outgrowth	39
38	Caudal corner of paraterga pointed from segment 3. Pleurosternal carinae particularly well-developed, surpassing rear contour until segment 16 or 17. Adenostyles often present on ♂ prefemora, pattern as in Fig. 19K. Gonopods as in Figs 20, 21	Tylopus degerboelae
–	Caudal corners of paraterga mainly narrowly rounded, pointed only from segment 15. Pleurosternal carinae less strongly developed	Tylopus pallidus
39	Coloration dark, brown. Sternal lamina between ♂ coxae 4 like a pair of separate, setiferous tubercles preceded by another pair of very small tubercles between coxae 3. Gonopod with lobe **l** devoid of an apical process	Tylopus asper
–	Coloration uniformly pale. Sternal lamina between ♂ coxae 4 single, not accompanied by additional tubercles in front or behind. Gonopod lobe **l** with a strong, apical, finger-shaped process	40
40	Mostly 3+3 tubercles at rear margin of metaterga. Sternal lamina between ♂ coxae 4 distinctly emarginate. Larger adenostyles close to midlength on ♂ postfemora and tibiae, femora with a distal knob. Gonopod process **h** slenderer and shorter, apex of lobule **m** not developed into a spine	Tylopus subcoriaceus
–	Mostly 4+4 tubercles at rear margin of metaterga. Sternal lamina between ♂ coxae trapeziform. Larger adenostyles on both postfemora and tibiae more distal, femoral knob missing. Gonopod process **h** unusually prominent, with a hook at base, apex of lobule **m** spiniform	Tylopus coriaceus

## Conclusion

Tylopus appears to be one of the largest millipede genera in Southeast Asia. The genus is best known from Thailand, which has 26 (> 63%) of the described species. With further progress in our knowledge of the millipede faunas of other, still poorly prospected, mostly neighbouring countries such as Laos, Myanmar, Cambodia and Vietnam, as well as southern China, the total of 41 Tylopus species can readily be expected at least to double. More congeners are likely to be found in Thailand as well. Golovatch and Enghoff (1993) attempted a preliminary phylogenetic analysis of Tylopus based on the 35 species then known, but given the incomplete state of our knowledge of Tylopus, we believe that a new phylogenetic analysis would be premature.

In Thailand, all Tylopus are confined to the northern, mountainous parts of the country (Map). Finding congeners south of Tak Province seems unlikely, but, since Tylopus are known also from all over Vietnam, including the southern parts of the country, this genus is likely to occur at least in the adjacent parts of Cambodia, from where no species have hitherto been recorded. At present the northern range limit of Tylopus lies in Yunnan Province, China, but it seems plausible that many more regions in southern China, even some north of Yunnan, might also prove to support Tylopus species. Since only a few species have been reported from Laos and Myanmar, another considerable increase in the number of congeners is more than likely after further collecting in those countries as well.

**Map:**
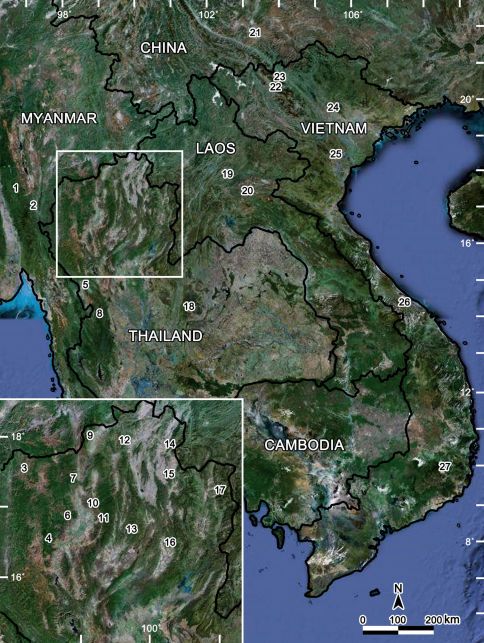
Distribution of Tylopus species: 2 species (Myanmar), 26 species (Thailand), 2 species (Laos), 1 species (China), 13 species (Vietnam): **1** Yado: Tylopus doriae (Pocock, 1895). **2** Village of Thao: Tylopus silvestris (Pocock, 1895). **3** Pha Mon Cave: Tylopus grandis sp. n. **4** Doi Inthanon: Tylopus affinis Golovatch & Enghoff, 1993, Tylopus allorugosus Golovatch & Enghoff, 1993, Tylopus asper Golovatch & Enghoff, 1993, Tylopus degerboelae Golovatch & Enghoff, 1993,Tylopus haplorugosus Golovatch & Enghoff, 1993, Tylopus jeekeli Golovatch & Enghoff, 1993, Tylopus perarmatus Hoffman, 1973, Tylopus prosperus Golovatch & Enghoff, 1993, Tylopus parajeekeli sp. n. **5** Ban Mussoe: Tylopus semirugosus Golovatch & Enghoff, 1993. **6** Doi Suthep: Tylopus affinis Golovatch & Enghoff, 1993, Tylopus allorugosus Golovatch & Enghoff, 1993, Tylopus baenzigeri Golovatch & Enghoff, 1993, Tylopus degerboelae Golovatch & Enghoff, 1993, Tylopus doriae (Pocock, 1895), Tylopus hoffmani Golovatch & Enghoff, 1993, Tylopus jeekeli Golovatch & Enghoff, 1993, Tylopus perarmatus Hoffman, 1973, Tylopus similirugosus Golovatch & Enghoff, 1993, Tylopus subcoriaceus Golovatch & Enghoff, 1993. **7** Doi Chiang Dao: Tylopus degerboelae Golovatch & Enghoff, 1993, Tylopus perarmatus Hoffman, 1973, Tylopus rugosus Golovatch & Enghoff, 1993. **8** Umphang District: Tylopus bispinosus sp. n. **9** Doi Pha Hom Pok: Tylopus amicus Golovatch & Enghoff, 1993, Tylopus pallidus Golovatch & Enghoff, 1993, Tylopus perplexus Golovatch & Enghoff, 1993, Tylopus poolpermorum Golovatch & Enghoff, 1993, Tylopus extremus sp. n. **10** BuathongWaterfall: Tylopus rugosus Golovatch & Enghoff, 1993. **11** Doi Phatang: Tylopus degerboelae Golovatch & Enghoff, 1993, Tylopus perarmatus Hoffman, 1973. **12** Ban Pang Rim Kon: Tylopus perarmatus Hoffman, 1973. **13** Thum Pha Thai: Tylopus perarmatus Hoffman, 1973. **14** Phucheefah: Tylopus perarmatus Hoffman, 1973. **15** Nam Min Waterfall: Tylopus perarmatus Hoffman, 1973. **16** Tham Pha Nang Khoi: Tylopus perarmatus Hoffman, 1973. **17** Ton Tong Waterfall: Tylopus veliger sp. n. **18** Phu Kheio: Tylopus coriaceus Golovatch & Enghoff, 1993, Tylopus pulvinipes Golovatch & Enghoff, 1993. **19** Luang Prabang: Tylopus nodulipes (Attems, 1953), Tylopus mutilatus (Attems, 1953). **20** XiengKuang: Tylopus mutilatus (Attems, 1953). **21** Mengzi County: Tylopus sinensis Golovatch, 1995. **22** Mt Fan-Si-Pan: Tylopus nodulipes (Attems, 1953). **23** O quy ho: Tylopus crassipes Golovatch, 1984, Tylopus maculatus Golovatch, 1984, Tylopus magicus Golovatch, 1984, Tylopus procurvus Golovatch, 1984. **24** Tam Dao: Tylopus strongylosomoides (Korsós & Golovatch, 1989), Tylopus tamdaoensis Korsós & Golovatch, 1989. **25** Cuc Phuong Nature Reserve: Tylopus granulatus Golovatch, 1984, Tylopus hilaroides Golovatch, 1984, Tylopus topali Golovatch, 1984. **26** Bana: Tylopus hilaris (Attems, 1937). **27** Peak Langbiang: Tylopus mutilatus (Attems, 1953).

Almost all Tylopus species are confined to forest habitats, especially montane ones. Most are local to highly local in distribution. There are only very few relatively widespread congeners, e.g. Tylopus doriae, Tylopus perarmatus or Tylopus degerboelae. At one locality, as many as nine congeners can co-occur, e.g. in Doi Inthanon and Doi Suthep mountains. This remarkable result indicates that many other high- to mid-montane forested areas in Indochina and southern China could support similarly rich faunules of Tylopus.

Within Tylopus sympatric groups, only Tylopus degerboelae appears to show a highly extended, almost annual pattern of seasonal activity, judging from the occurrence of adults of both sexes at Doi Inthanon and, especially, Doi Suthep. Adults of the bulk of congeners living at either (four species each) or both (five species) of these mountain ranges tend to be autumnal, their collection being confined to September to November. This probably means that these species represent a single, autumnal phenofauna. The sole, possibly noteworthy exception is Tylopus asper which has heretofore been found only at Doi Inthanon and only in May. Whether this species represents a different phenofauna or not, remains open to question. Special observations are required to reveal the phenology and breeding seasons of Tylopus at least in northern, mostly montane Thailand.

## Supplementary Material

XML Treatment for Tylopus bispinosus

XML Treatment for Tylopus grandis

XML Treatment for Tylopus extremus

XML Treatment for Tylopus veliger

XML Treatment for Tylopus parajeekeli

XML Treatment for Tylopus allorugosus

XML Treatment for Tylopus degerboelae

XML Treatment for Tylopus haplorugosus

XML Treatment for Tylopus jeekeli

XML Treatment for Tylopus prosperus

XML Treatment for Tylopus rugosus

XML Treatment for Tylopus perarmatus
